# Multi-omic analysis of the cardiac cellulome defines a vascular contribution to cardiac diastolic dysfunction in obese female mice

**DOI:** 10.1007/s00395-023-00983-6

**Published:** 2023-03-29

**Authors:** Malathi S. I. Dona, Ian Hsu, Alex I. Meuth, Scott M. Brown, Chastidy A. Bailey, Christian G. Aragonez, Jacob J. Russell, Crisdion Krstevski, Annayya R. Aroor, Bysani Chandrasekar, Luis A. Martinez-Lemus, Vincent G. DeMarco, Laurel A. Grisanti, Iris Z. Jaffe, Alexander R. Pinto, Shawn B. Bender

**Affiliations:** 1 Baker Heart and Diabetes Research Institute, 75 Commercial Rd Prahran, Melbourne, VIC 3004 Australia; 2 Biomedical Sciences, University of Missouri, E102 Vet Med Bldg, Columbia, MO USA; 3 Dalton Cardiovascular Research Center, University of Missouri, Columbia, MO USA; 4 Research Service, Harry S Truman Memorial Veterans Hospital, Columbia, MO USA; 5 Medicine, University of Missouri School of Medicine, Columbia, MO USA; 6 Medical Pharmacology and Physiology, University of Missouri School of Medicine, Columbia, MO USA; 7 Molecular Cardiology Research Institute, Tufts Medical Center, Boston, MA USA; 8 Centre for Cardiovascular Biology and Disease Research, La Trobe University, Melbourne, Australia

**Keywords:** Obesity, Mineralocorticoid receptors, Coronary, Single-cell RNA sequencing, Inflammation

## Abstract

Coronary microvascular dysfunction (CMD) is associated with cardiac dysfunction and predictive of cardiac mortality in obesity, especially in females. Clinical data further support that CMD associates with development of heart failure with preserved ejection fraction and that mineralocorticoid receptor (MR) antagonism may be more efficacious in obese female, versus male, HFpEF patients. Accordingly, we examined the impact of smooth muscle cell (SMC)-specific MR deletion on obesity-associated coronary and cardiac diastolic dysfunction in female mice. Obesity was induced in female mice via western diet (WD) feeding alongside littermates fed standard diet. Global MR blockade with spironolactone prevented coronary and cardiac dysfunction in obese females and specific deletion of SMC-MR was sufficient to prevent obesity-associated coronary and cardiac diastolic dysfunction. Cardiac gene expression profiling suggested reduced cardiac inflammation in WD-fed mice with SMC-MR deletion independent of blood pressure, aortic stiffening, and cardiac hypertrophy. Further mechanistic studies utilizing single-cell RNA sequencing of non-cardiomyocyte cell populations revealed novel impacts of SMC-MR deletion on the cardiac cellulome in obese mice. Specifically, WD feeding induced inflammatory gene signatures in non-myocyte populations including B/T cells, macrophages, and endothelium as well as increased coronary VCAM-1 protein expression, independent of cardiac fibrosis, that was prevented by SMC-MR deletion. Further, SMC-MR deletion induced a basal reduction in cardiac mast cells and prevented WD-induced cardiac pro-inflammatory chemokine expression and leukocyte recruitment. These data reveal a central role for SMC-MR signaling in obesity-associated coronary and cardiac dysfunction, thus supporting the emerging paradigm of a vascular origin of cardiac dysfunction in obesity.

**Supplementary Information:**

The online version contains supplementary material available at 10.1007/s00395-023-00983-6.

## Introduction

Coronary microvascular dysfunction (CMD) is independently predictive of cardiac morbidity and mortality in people with obesity and diabetes [46]. Importantly, obesity is more common in women and females are more likely than males to develop CMD rather than obstructive coronary artery disease [57]. While premenopausal women are protected from heart disease relative to men, that protection is lost in females with obesity and metabolic dysfunction [1]. A recent report revealed an association of CMD and cardiac diastolic dysfunction in females, but not males, with obesity and diabetes [27]. This association has significant implications for outcomes since patients with both CMD and diastolic dysfunction have a > fivefold increased risk of heart failure with preserved ejection fraction (HFpEF) hospitalization versus patients with isolated CMD or diastolic dysfunction [58]. Accordingly, it has been suggested that obesity-associated CMD may be a primary mechanism of cardiac diastolic dysfunction and HFpEF [30, 47, 50], conditions for which treatments are limited. While cardiovascular disease mortality is declining in males, it is rising in middle-aged premenopausal females [59, 65]; thus, delineation of mechanisms of CMD in females with obesity and metabolic disease is a critical unmet medical need [49].

Recent evidence, from us and others, revealed that mineralocorticoid receptor (MR) blockade attenuates obesity and diabetes-associated CMD in preclinical models [4, 7] and patients [19, 33] independent of blood pressure. Further, pharmacologic MR antagonism with spironolactone (Spiro) prevented obesity-associated diastolic dysfunction in female mice [6] consistent with a recent post hoc analysis of the Treatment of Preserved Cardiac Function Heart Failure with an Aldosterone Antagonist (TOPCAT) trial suggesting greater benefit of MR blockade in obese female HFpEF patients compared to males [43]. Mechanistically, SMC dysfunction (i.e., hypercontractility) has been found to precede endothelial dysfunction in obesity [22] suggesting a potential role for SMC-MR signaling to contribute to impaired coronary and cardiac function in obese females via crosstalk between SMC and other cells in the heart. Indeed, deletion of SMC-MR abrogates aging-associated arteriolar hypercontractility and hypertension [15, 40]. The role of SMC-MR in obesity-associated coronary and cardiac dysfunction has not been explored. To that end, we hypothesized that SMC-MR deletion would prevent both CMD and cardiac diastolic dysfunction in obese female mice thereby expanding our mechanistic understanding of these common conditions.

To test this hypothesis, we induced obesity in female SMC-MR knockout (SMC-MR-KO) and MR-Intact littermate mice by feeding a high-fat/high-carbohydrate western diet (WD) for 16 weeks. Additional studies were conducted in obese mice treated with and without Spiro. We quantified cardiac diastolic function and characterized the coronary and cardiac phenotype compared to SMC-MR-KO and MR-Intact mice fed control diet. We further determined gene expression changes in the non-cardiomyocyte fraction of the mouse heart in response to WD in the presence or absence of SMC-MR utilizing single-cell RNA sequencing (scRNA-seq). We show that SMC-MR deletion protects females from obesity-induced cardiac and coronary dysfunction and scRNA-seq analysis revealed novel cell-specific gene expression changes induced by obesity that are modified by SMC-MR. These findings support the emerging concept of a vascular origin of cardiac diastolic dysfunction in obesity.

## Methods

### Animals

All animal protocols were approved by the Institutional Animal Care and Use Committees of the Harry S Truman Memorial Veterans Hospital and the University of Missouri in compliance with the *Guide for the Care and Use of Laboratory Animals* (National Institutes of Health). All animals were housed in a temperature-controlled room (12:12-h light–dark cycle) and provided ad libitum access to water and either a standard control diet (Con; LabDiet 5008) or a western diet (WD; TestDiet 58Y1 modified) consisting of 46% fat and 36% carbohydrate (17.5% each from sucrose and high fructose corn syrup) for 16 weeks beginning at 14–24 weeks of age. Two experimental paradigms were utilized. First, female C57BL/6J mice (Jackson Labs) were randomly divided into 3 groups: Con, WD, or WD treated with the MR antagonist spironolactone (Spiro, sc, 0.63 mg·day^−1^; Innovative Research of America) for 16 weeks. Con and WD-fed mice received placebo pellets for 16 weeks. Second, mice with inducible MR deletion specifically in smooth muscle cells (SMC-MR knockout [SMC-MR-KO] mice) were generated, as previously described [15, 40], by crossing floxed MR mice with mice containing a Cre-recombinase-ER^T2^ gene driven by the smooth muscle actin promoter (SMA-Cre-ER^T2^) and activated by tamoxifen. These mice are compared with floxed MR/SMA-Cre-ER^T2^ negative (MR-intact) littermates. Both strains were treated with tamoxifen at 6–8 weeks of age, resulting in MR deletion in the Cre positive animals as previously confirmed [15, 40]. SMR-MR-KO and MR-intact mice were fed Con and WD for 16 weeks.

### Plasma and urine parameters

Twenty-four hour urine collection was performed during the final week of diet feeding as was glucose tolerance testing. Following measurement of fasting blood glucose, mice were injected with glucose (ip, 1 g/kg) and blood glucose subsequently measured at 15, 30, 60, and 120 min post-injection via glucometer (AlphaTRAK 2, Zoetis). On the day of euthanasia, mice were fasted for 5 h and anesthetized with isoflurane (2–4% in 100% O_2_); blood was collected via the inferior vena cava, processed to plasma, and frozen at – 80 °C. Blood glucose was determined by glucometer. Plasma aldosterone was quantified by radioimmunoassay (Tecan MG13051). All other plasma and urine measures were analyzed at Comparative Clinical Pathology Services (Columbia, MO).

### Echocardiography

During the final week of diet feeding/treatment, transthoracic echocardiography was performed (Vevo2100, FUJIFILMS, Visualsonics, Toronto) with an MS400 high frequency echo probe at the Small Animal Ultrasound Imaging Center at the Harry S Truman Memorial Veterans Hospital. Under anesthesia (0.75–4% isoflurane in 100% O_2_), mice were placed on a heated platform to maintain body temperature at 37 °C and two-dimensional echocardiograms were performed in the apical four chamber view. Initially, a small sample volume was positioned in the left ventricle (LV) just proximal to the mitral leaflets to acquire early (E) and late (A) diastolic blood flow velocities in pulse wave (PW) Doppler mode. Isovolumic relaxation time (IVRT) was also determined from the PW spectra. B- and M-mode images of the LV and septum in short axis view were acquired at the level of the papillary muscles. Left ventricular posterior and septal wall thicknesses at end diastole (LV PWTd and LV SWTd), luminal diameters (LVIDs and LVIDd), and ejection fraction (EF) were determined offline in M-mode. B-mode images in modified long axis (ascending aortic) view were acquired for determination of left atrial and aortic diameters. Next, Tissue Doppler Imaging (TDI) was performed in the apical four chamber view to acquire early (E’) and late (A’) septal annular velocities. Parameters were assessed using an average of three beats from three different spectra, and calculations were made in accordance with the American Society of Echocardiography guidelines as well as specific guidelines for rodent echocardiography. All data were acquired and analyzed offline by a single blinded observer.

### Aortic vascular measurements and blood pressure

During the final week of diet feeding/treatment, in vivo aortic stiffness was evaluated in isoflurane-anesthetized mice (1.75% in 100% O_2_) by pulse wave velocity (PWV) using Doppler ultrasound (Indus Mouse Doppler System, Webster, TX), as previously described [13]. Briefly, using the transit time method, PWV was quantified as the difference in arrival times of a Doppler pulse wave at two locations along the aorta at a fixed distance [29]. The distance between the two locations along the aorta is divided by the difference in arrival times and is expressed in m/s. Velocity waveforms were acquired at the aortic arch followed immediately by measurement at the descending aorta 35 mm distal to the aortic arch. Systolic blood pressure was determined by tail-cuff plethysmography (BP-2000, Visitech) during the final week of treatment, as previously described [4].

### Coronary vasomotor function

Following blood collection under anesthesia (2–4% in 100% O_2_), mice were perfused via the left ventricle at 100 mmHg with ice-cold physiological salt solution (PSS) comprised of (in mM): 119 NaCl, 4.7 KCl, 2.5 CaCl·2H_2_O, 1.17 MgSO_4_·7H_2_O, 1.18 KH_2_PO_4_, 0.027 EDTA, 25 NaHCO_3_, and 5.5 glucose (pH 7.4). The heart was subsequently removed and segments of the left coronary artery (~ 1 mm) dissected and mounted on 17 µm stainless steel wires in oxygenated PSS (95% O_2_–5% CO_2_) in a small vessel myograph for isometric tension recording (Danish Myo Technology, Aarhus, Denmark), as previously described [2, 3]. Vessel length was quantified after mounting with a calibrated ocular micrometer. Following equilibration and normalization using an established procedure [52], vessel viability was confirmed by exposure to 80 mM KCl PSS. Following washing, vasoconstrictor responses to the thromboxane A_2_ analog U46619 (10 nM–1 µM) were assessed as well as vasodilator responses to acetylcholine (ACh; 1 nM–0.1 mM) and sodium nitroprusside (SNP; 1 nM–0.1 mM) following preconstriction with U46619 (100–300 nM). A subset of vessels were pretreated with the superoxide dismutase mimetic Tempol (1 mM for 20 min) prior to assessment of ACh-induced vasodilation. Vasodilator responses are reported as percent maximal dilation from U46619 preconstriction. Vasoconstrictor responses are reported as developed tension normalized to vessel length (mN/mm). Since this vessel does not develop spontaneous myogenic tone, minimum tension (i.e., maximal dilation) was determined following the normalization procedure.

### Atomic force microscopy

Aortic endothelial cortical stiffness was quantified on* en face* preparations, as previously described [8]. Briefly, the endothelial surface of a section of thoracic aorta (~ 2 mm) was exposed by opening the vessel longitudinally and fastening the section to a plastic cover slip with Cell-Tak. Endothelial stiffness was then assessed via a cell nanoindentation protocol with an atomic force microscope.

### RT-PCR

Aortas and periovarian adipose tissue were homogenized in a Tissue-Lyser (Qiagen) and total RNA was extracted using the Qiagen RNeasy Fibrous (aorta) or Lipid (adipose) Tissue kit and quantified using a Nanodrop spectrophotometer (Thermo Scientific). First-strand cDNA was synthesized from total RNA using the Improm-II reverse transcription kit (Promega) and quantitative real-time PCR was performed using the CFX Connect Real-Time PCR Detection System (Biorad) using target specific primers (Online Resource 1). PCR reactions using iTaq Universal SYBR Green SMX (Biorad), thermal conditions, and melt curve analysis were performed as previously described [4]. GAPDH was used as an internal control gene and messenger RNA (mRNA) expression values were calculated based on cycle thresholds (CTs) via the 2^ΔΔCT^ method, where ΔCT = GAPDH CT – gene of interest CT and are presented normalized to control mice, which were set at 1.

### Immunohistochemistry and staining

Left ventricular tissue was immersion fixed in 10% buffered formalin, dehydrated in ethanol, paraffin embedded, and sectioned in 5 µm slices. To evaluate fibrosis, sections were stained with picrosirius red (PR) for determination of cardiac interstitial and periarterial collagen. Images were obtained using an EVOS FL Auto Imaging System and quantified using the thresholding function in ImageJ. Periarterial fibrosis was quantified as the ratio of PR-stained periarterial area to luminal circumference. Interstitial fibrosis was quantified as the percent area of myocardial PR staining. Cardiac capillary density was quantified in FITC-conjugated CD31 (1:50, Novus)-stained cardiac sections. In additional sections, following sodium citrate buffer antigen retrieval, hearts were blocked with 10% FBS in PBS and 0.3% H_2_O_2_ to prevent endogenous peroxide activity. Hearts were incubated with antibodies against CD3 (1:100; Abcam #ab5690), CD68 (1:100; Abcam #ab31630), or mast cell tryptase (1:100; Abcam #ab2378). Washed slides were incubated with the appropriate HRP conjugated secondary antibody. Stained hearts were developed using a DAB Substrate Kit (Thermo Fisher Scientific) and conjugated with hematoxylin to identify nuclei. Staining was visualized on a Nikon Eclipse microscope at 20X magnification and analyzed using ImageJ from 10 fields per heart. Additional immunofluorescence studies were performed to assess VCAM-1 and Cyp1a1 protein expression in coronary vessels and coronary endothelium, respectively. Briefly, following antigen retrieval (heat and sodium citrate buffer) and blocking non-specific protein binding (Abcam #ab64226), sections were incubated with smooth muscle α-actin (1:500; Novus #nb300-978) antibody in conjunction with antibodies against either VCAM-1 (1:100; Abcam #ab134047) or Cyp1a1 (1:100; Proteintech #13241-1-ap) at 4 °C overnight. Slides were then washed and incubated with Alexa Fluor 488 (1:200; ThermoFisher #A-11055) and Alexa Fluor 647 (1:200; ThermoFisher #A-21443) conjugated secondary antibodies after which slides were washed once more and mounted with antifade reagent. Fluorescence was detected on a Leica DMI4000 B confocal microscope and analyzed using ImageJ software. Specifically, vessels were identified by smooth muscle α-actin staining with subsequent quantification of VCAM-1 staining within the α-actin stained region and endothelial Cyp1a1 staining inside the luminal border of the α-actin stained region.

### Cardiac RNA-Seq

High-throughput sequencing of LV was performed at the University of Missouri DNA Core Facility. Briefly, LV tissue was homogenized in a Tissue-Lyser (Qiagen) and total RNA was extracted using the Qiagen RNeasy Lipid Tissue kit and quantified using a Nanodrop spectrophotometer (Thermo Scientific). Libraries were constructed following the manufacturer’s protocol using the Illumina TruSeq mRNA stranded sample preparation kit. The RNA input concentration was determined using the Qubit HS RNA assay kit and Qubit fluorometer (Invitrogen) and RNA quality assessed using the Fragment Analyzer automated electrophoresis system (Agilent). Briefly, the poly-A containing mRNA is purified from total RNA, fragmented, double-stranded cDNA is generated from fragmented RNA, the index containing adapters are ligated, and the amplified cDNA constructs were purified by addition of AxyPrep Mag PCR Clean-up beads. The final construct of each purified library was evaluated using the Fragment Analyzer, quantified using the Qubit HS dsDNA assay kit and fluorometer, and diluted according to Illumina’s standard sequencing protocol for sequencing on the NextSeq 500 via single end 75 base pair reads.

RNA-Seq data were processed and analyzed as previously described [20]. Briefly, latent Illumina adapter sequence was identified and removed from input 100-mer RNA-Seq data using Cutadapt. Subsequently, input RNA-Seq reads were trimmed and filtered to remove low quality nucleotide calls and whole reads, respectively, using the Fastx-Toolkit. To generate the final set of quality-controlled RNA-Seq reads, foreign or undesirable sequences were removed by similarity matching to the Phi-X genome (NC_001422.1), the relevant ribosomal RNA genes as downloaded from the National Center for Biotechnology Information, or repeat elements in RepBase, using Bowtie. This final set of quality-controlled RNA-Seq reads was aligned to the Ensemble *Mus musculus* genome sequence, GRCm38.p5, using STAR with the default settings, which also generated the initial expression estimates for each annotated gene. The R Bioconductor package DESeq2 was used to normalize the gene expression estimates across the samples and to analyze the differential expression of genes between sample types. Potential outlier samples were identified and removed at this stage using a combination of Principle Component Analysis and the R libraries nortest and outliers. Gene expression estimates were recalculated after outliers were removed. A gene was identified as being differentially expressed between two conditions when the FDR-corrected *p* value of its expression ratio was less than 0.05. Subsequent data were reformatted, sorted and filtered using a variety of R commands and Bash command-line scripts, which are available upon request. Ingenuity Pathway Analysis (IPA; Qiagen) was utilized for examination of the top differentially up- and downregulated genes and the corresponding top networks, pathways, and associated biological processes.

### Cardiac non-cardiomyocyte single-cell preparation

Single-cell suspensions from isolated mouse hearts were prepared, as previously described [54]. Briefly, mice were euthanized with isoflurane and the heart exposed via bilateral thoracotomy before perfusion with DPBS (0.8 mM CaCl_2_; 10 min). Hearts were subsequently isolated, the atria, valves, and right ventricle removed, and the LV minced to ~ 1 mm cubes. Minced LV tissue was digested in perfusion buffer containing collagenase IV (2 mg/ml; Worthington Biochemical) and Dispase II (1.2 units/ml; Sigma-Aldrich) for 45 min at 37 °C with suspension trituration every 15 min using 1000 µl micropipettes. The resulting cell suspension was filtered through a 70 µm filter, diluted in 15 ml perfusion buffer, and pelleted at 200 rcf for 20 min at 4 °C with centrifuge brakes disengaged. Cell supernatant was aspirated, the pellet resuspended in perfusion buffer, and re-pelleted as described above. The resulting cell pellets were resuspended in FACS buffer (HBSS, 2% FBS; Gibco), passed through a 40 µm filter, pelleted as described above, and resuspended in FACS buffer for subsequent staining and cell sorting. Intact, nucleated non-myocyte cells were subsequently isolated via flow cytometry after staining with Vybrant DyeCycle Ruby nuclear stain (10 µM; ThermoFisher V10273) and SYTOX Green viability stain (30 nM; ThermoFisher S7020).

### Single-cell RNA library preparation and sequencing

Libraries were constructed by following the manufacturer’s protocol with reagents supplied in 10x Genomics Chromium Next GEM Single Cell 3′ Kit v3.1. Briefly, cell suspension concentration and viability were measured manually and with an Invitrogen Countess II automated cell counter. Cell suspension (900 cells per microliter), reverse transcription master mix, and partitioning oil were loaded on a Chromium Next GEM G chip with a cell capture target of 5000 cells per library. Post-Chromium controller GEMs were transferred to a PCR strip tube and reverse transcription performed on an Applied Biosystems Veriti thermal cycler at 53 °C for 45 min. cDNA was amplified for 12 cycles and purified using Axygen AxyPrep MagPCR Clean-up beads. cDNA fragmentation, end-repair, A-tailing and ligation of sequencing adaptors was performed according to manufacturer specifications. The final library was quantified with the Qubit HS DNA kit and the fragment size was analyzed using an Agilent Fragment Analyzer system. Libraries were pooled and sequenced on an Illumina NovaSeq to generate 50,000 reads per cell with a sequencing configuration of 28 base pair (bp) on read1 and 98 bp on read2. Isolation of single cells was performed in three batches on separate days with samples from each treatment group included each day to mitigate batch effects.

### Analysis of single-cell RNA-Seq data

The raw sequencing data were processed using Cell Ranger version 3.1.0 (10x Genomics) before the subsequent analysis. Cell Ranger pipeline used fastq files and aligned sequencing reads to the mm10 transcriptome version 3.0.0 to quantify the expression of genes in each cell. This resulted in data for 83,669 cells that passed quality control steps implemented in Cell Ranger. The filtered count data matrices obtained from cell ranger software were then used for the subsequent analysis. Analyses of scRNA-seq processed data were performed in R version 3.6 and 4.0.1 (https://www.R-project.org/) using Seurat suite versions 3.0 [56] and tidyverse [64] packages. Further quality control measures of cells with < 200 or > 8000 expressed genes and genes that were expressed in less than 3 cells were applied per sample manner. In addition, cells with more than 30% of UMI mapping to mitochondrial genes were filtered out to control dead or damaged cells. These steps further removed 298 cells from the analysis. The final dataset contains 83,371 cells from 12 mice in 4 conditions and gene expression information for 19,905 genes.

Dimensionality reduction was performed using principal component analysis (PCA) to explore transcriptional heterogeneity and clustering. PC loading for 40 PCs were used as input for a graph-based clustering approach to cluster cells with clustering resolution 0.8. Cells and clusters were visualized on a t-distributed stochastic neighbor embedding (t-SNE) two-dimensional plot generated using the same PC loadings used for the clustering. To optimize the t-SNE plot, 1000 iterations and 289 perplexities were used. The identified cell clusters were then annotated based on known marker genes. Figures were primarily generated using Seurat and ggplot2 R packages (https://ggplot2-book.org/).

### Differential expression analysis

The differential expression (DE) analysis was performed for each cell population separately. To identify DE genes between groups, we first identified genes expressed in at least 10% of cells in at least one of the groups being compared. We then used MAST R package version 1.12.0 [17] to perform DE testing method MASTcpmDetRate considering the cellular detection rate as a covariate. A threshold of uncorrected *p* < 0.01 was used to define statistically significant DE genes between groups.

### Gene ontology analysis

Gene Ontology (GO) over-representation analysis for differentially expressed gene lists (uncorrected *p* < 0.01) was performed using the enrichGO function from clusterProfiler R package version 3.16.1 [67]. The R package org.Mm.eg.db: Genome wide annotation for Mouse, R package version 3.11.4 [9] was used to obtain all gene ontology mappings. The over-representation of GO Biological Process terms (GO-BP) was calculated using the entire list of genes identified in the experiment as the background gene list for *Mus musculus* with minimum and maximum gene set sizes 10 and 500, respectively. The similarity between enriched GO-BP terms were calculated using the simplify R function from clusterProfiler R package. GO-BP terms with semantic similarity more than 0.7 were treated as redundant terms and discarded from the analysis. The Benjamini–Hochberg adjusted *p* value cutoff of 0.05 was used to determine statistically significant GO-BP terms.

### Flow cytometry

Immune cells were isolated from the heart by enzymatic digestion, as previously described [23]. Briefly, hearts were isolated, flushed with HBSS, and manually digested into ~ 1 mm^3^ pieces. Heart pieces were then enzymatically digested in collagenase II (150 U/mL; Worthington Biochemical) and trypsin (0.6 mg/mL; Worthington Biochemical) at 37 °C with agitation. Following digestion, myocyte and non-myocyte fractions were separated by centrifugation at 8 × g for 5 min. The non-myocyte containing supernatant was passed through a 70 µm cell strainer prior to flow cytometry analysis.

Cells were stained in 1% FBS in PBS for 30 min at 4 °C with the following antibodies: LIVE/DEAD Fixable Aqua Dead Cell Stain Kit (1:40, Invitrogen #L34957), CD3-PE-Cy7 (1:100; Biolegend #100220), CD4-PE (1:100; BD Biosciences #5530449), CD11b-FITC (1:200; Biolegend #101206), CD68-PE (1:50; Biolegend #137014), CD45-BV480 (1:100; BD Biosciences #746682), CD80-PE-Cy7 (1:100; Biolegend #104712), CD117-APC-H7 (1:100; BD Biosciences #560185) and CD196-BV711 (1:100; BD Biosciences #740648). Positive staining was identified based on single antibody controls which were performed for all antibodies on all tissues examined and fluorescence minus one controls were performed on splenic samples to validate cell staining. Isotype controls were also performed on splenic samples using PE-Cy7 rat IgG2b (κ isotype, 1:100, Biolegend 400617), PE rat IgG2a (κ isotype, 1:100, Biolegend 400507), FITC rat IgG2b (κ isotype, 1:100, Biolegend 400633), BV480 rat IgG2b (κ isotype, 1:100, BD Biosciences #565649), BV711 rat IgG2b (κ isotype, 1:100, BD Biosciences #563045) and APC-H7 rat IgG2b (κ isotype, 1:100, BD Biosciences #560200). Following staining, cells were washed twice with PBS and analyzed by flow cytometry using a BD LSRFortessa X-20. Analysis was performed in FlowJo software.

### Cardiac cytokine analysis

Quantitative proteomic analysis of cardiac cytokines was performed on whole left ventricular lysates by RayBiotech (Mouse Cytokine Array Q4000) and statistical differences between groups were assessed by Wilcoxon test.

### Data analysis and statistics

Data are presented as mean ± standard error with individual data points shown, when appropriate. Statistical analysis was performed using Student *t*-test for planned comparisons, two-way analysis of variance (for repeated measures, when appropriate) with Fisher least significant difference post hoc analysis, as appropriate, in SigmaPlot (SyStat) or Prism (Graphpad). A *p* value ≤ 0.05 was considered significant

## Results

**Fig. 1:**
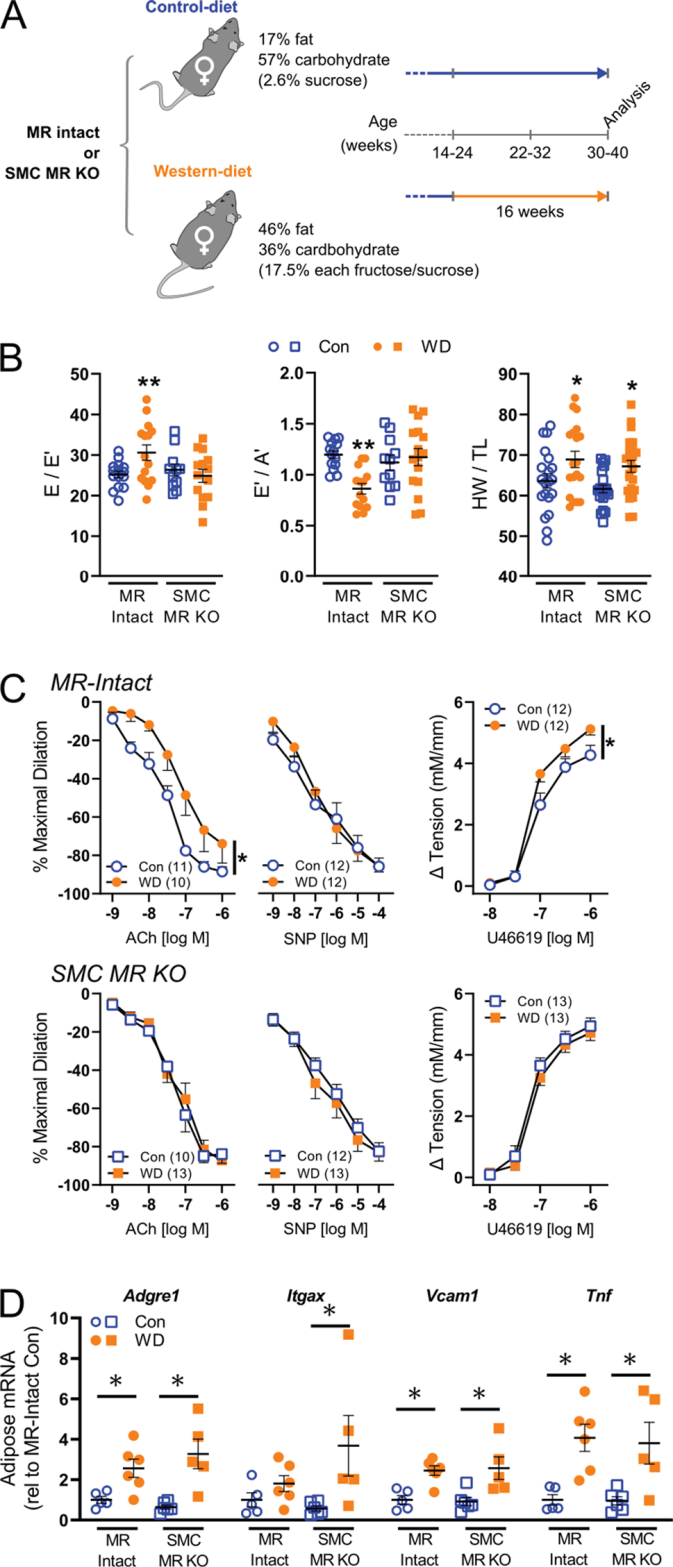
Smooth muscle cell mineralocorticoid receptor knockout (SMC-MR-KO) prevents cardiac diastolic and coronary vascular dysfunction in western diet (WD)-fed female mice independent of adipose inflammation. **a** Mouse cohorts and experimental conditions of the study. All mice were analyzed 16 weeks after Western diet (WD) or control diet (Con) feeding at 30–40 weeks of age. **b** Indices of cardiac diastolic function, specifically estimated left ventricular filling pressure (E/E’) and early-to-late diastolic septal annulus motion ratio (E’/A’), and cardiac weights (heart weight-to-tibia length ratio; HW/TL) in control (Con) and WD-fed mice. **c** Vasodilator responses of isolated coronary arteries to endothelium-dependent (acetylcholine, ACh), -independent (sodium nitroprusside, SNP) agonists as well as vasoconstrictor responses to the thromboxane A_2_ analog U46619. **d** Expression of inflammatory genes in reproductive adipose tissue. Values are mean ± SE with individual data points shown (**b**, **d**). *p < 0.05 versus Con or comparison indicated; **p < 0.05 versus all other groups

*Systemic MR blockade and SMC-specific MR deletion do not impact traditional cardiac risk factors in obese female mice.* WD feeding-induced phenotypic changes in female mice were unchanged by MR blockade with Spiro (Online Resource 2), as we previously reported [6, 32], nor by SMC-specific MR deletion (Fig. 1A, Online Resource 3). Specifically, WD-induced increases of blood glucose, plasma insulin, plasma aldosterone, and plasma cholesterol as well as WD-induced proteinuria and increased urinary blood urea nitrogen (BUN) levels were unchanged by SMC-MR deletion (Online Resource 3). Similar to a recent report [45], SMC-MR-KO mice fed WD exhibited a modest (~ 10%) reduction in average body weight and reduced periovarian adipose weight, compared to MR-Intact controls fed WD (Online Resource 3), with no change in glucose intolerance (Online Resource 4).

*Systemic MR blockade and SMC-specific MR deletion prevent WD-induced cardiac diastolic and coronary vascular dysfunction independent of blood pressure and adipose inflammation.* WD feeding impaired cardiac diastolic function, indicated by increased LV filling pressure (E/E’), reduced septal wall motion in diastole (E’/A’), left atrial distension (LA/Ao), and induced cardiac hypertrophy in female mice (Fig. 1B, Online Resources 5-7). Results herein extend prior work by demonstrating concomitant impairment of coronary endothelium-dependent vasodilation with WD feeding in female mice. Furthermore, MR blockade with Spiro attenuated both the coronary endothelial and cardiac dysfunction, but not the cardiac hypertrophy, induced by WD feeding (Online Resources 5 and 6). Additional mechanistic studies revealed that SMC-specific MR deletion in female mice is sufficient to reproduce the benefits of global MR inhibition in obese females (Fig. 1). Indeed, unlike WD-fed MR-Intact mice, SMC-MR-KO mice fed a WD did not exhibit impaired diastolic function despite similar WD-induced cardiac hypertrophy (Fig. 1B, Online Resource 7). Further, impaired endothelium-dependent vasodilation and enhanced vasoconstriction to the thromboxane analog U46619 induced by WD feeding in MR-Intact mice was absent in WD-fed SMC-MR-KO mice (Fig. 1C). There were no differences in diameters of coronary vessels studied (Online Resource 8). Importantly, blood pressure and aortic pulse wave velocity (i.e., aortic stiffness) were not changed by either WD feeding or SMC-MR deletion (Online Resource 3). Lastly, WD-induced visceral adipose inflammation indicated by increased gene expression of *Adgre1*, *Itgax*, *Vcam1*, and *Tnf*, was unchanged by SMC-MR-KO mice (Fig. 1D).

**Fig. 2:**
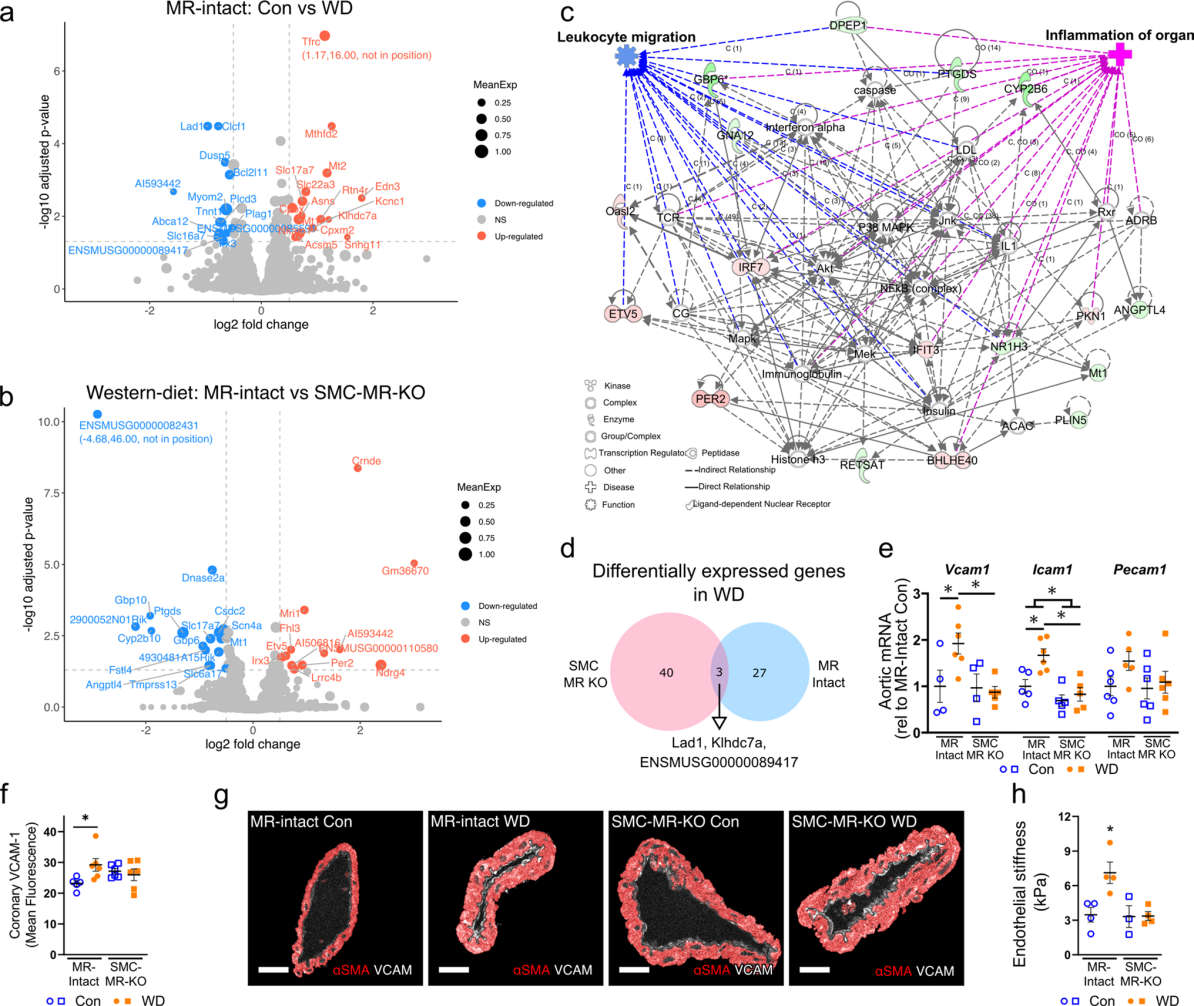
Smooth muscle cell mineralocorticoid receptor knockout (SMC-MR-KO) alters western diet (WD)-induced changes of the cardiac transcriptome. **a** Analysis of cardiac transcripts (13,598 transcripts) revealed differential expression of 30 transcripts induced by WD feeding in MR-Intact mice. Blue and red circles indicated genes down- or upregulated after WD (14 downregulated, blue dots; 16 upregulated, red dots). **b** Differential gene expression in WD-fed SMC-MR-KO mice versus WD-fed MR-Intact mice (16 downregulated, blue dots; 12 upregulated, red dots). Colored circles in volcano plots (panels a and b) indicate genes with log2 fold change > 0.5 and corrected *p* < 0.05. **c** Top differentially regulated IPA network (IPA score = 42; green nodes, downregulated; red nodes, upregulated) in WD-fed SMC-MR-KO versus WD-fed MR-Intact mice with enriched relevant biological processes. **d** WD feeding induced unique transcriptomic signatures in WD-fed MR-Intact and SMC-MR-KO mice (3 overlapping differentially expressed [log2 fold change > 0.5, corrected *p* < 0.05] transcripts). **e** Gene expression of adhesion molecules in whole aortic tissue from each group. **f** Expression of VCAM-1 in coronary vessels by immunofluorescence and **g** representative images by group. **h** Aortic endothelial cortical stiffness assessed by atomic force microscopy. Values are mean ± SE with individual data points shown; *p < 0.05 from all other groups or noted comparison

*SMC-specific MR deletion shifts WD-induced changes in the cardiac transcriptome.* Since SMC-MR deletion did not change traditional risk factors, we explored local cardiac-specific changes associated with SMC-MR signaling in the setting of WD feeding. Specifically, we performed bulk RNA sequencing of LV tissues and examined the unique cardiac transcriptomic signatures induced by WD feeding (Fig. 2A; Online Resource 9) and how the transcriptome differed with SMC-MR deletion in the setting of WD feeding (i.e., compared to WD-fed MR-Intact; Fig. 2B). Differential gene expression analysis did not reveal any genes/pathways altered as a result of SMC-MR-KO (compared to MR-Intact) in control chow-fed mice (Online Resource 10); however, key pathways that were identified using gene ontology (GO) enrichment analysis across other group comparisons included: water homeostasis (downregulated in WD-fed versus control-fed MR-Intact mice), circadian regulation (upregulated in WD-fed SMC-MR-KO WD versus WD-fed MR-Intact mice), and ketone metabolism (downregulated in WD-fed SMC-MR-KO versus WD-fed MR-Intact mice) (Online Resource 10). Changes in water homeostasis and circadian regulation are consistent with established associations of MR in regulating these biological processes. Moreover, Ingenuity Pathway Analysis (IPA) analysis of differentially expressed genes in WD-fed versus control-fed MR-Intact mice, revealed enrichment of ‘hypertrophy’ (consistent with increased HW/TL with WD feeding) and ‘quantity of reactive oxygen species (ROS)’ biological processes in the top gene network (Online Resource 11). Accordingly, increased ROS in WD-fed MR-Intact mice was confirmed by restoration of coronary endothelium-dependent vasodilation by the superoxide dismutase mimetic Tempol (Online Resource 11). Further, in WD-fed SMC-MR-KO compared to WD-fed MR-Intact mice, the top gene network was enriched for biological processes including ‘inflammation of organ’ and ‘leukocyte migration’ (Fig. 2C). Interestingly, WD-induced cardiac transcriptomic changes were unique across genotypes with only 3 shared genes (Fig. 2D). Directional gene changes in these IPA pathways in WD-fed SMC-MR-KO mice were generally consistent with reduced inflammation and leukocyte migration. Accordingly, assessment of aortic adhesion molecule gene expression demonstrated upregulation of *Vcam1* and *Icam1* in WD-fed MR-Intact mice indicative of vascular inflammation that is prevented in WD-fed SMC-MR-KO mice (Fig. 2E). Assessment of coronary VCAM-1 protein expression with immunofluorescence revealed increased VCAM-1 staining in WD-fed MR-Intact mice compared to Con-fed MR-Intact mice that was prevented in WD-fed SMC-MR-KO mice (Fig. 2F and G). Lastly, atomic force microscopy revealed that WD-fed MR-Intact mice exhibited increased aortic endothelial cortical stiffness, a marker of vascular injury [37], that was prevented in WD-fed SMC-MR-KO mice (Fig. 2H).

**Fig. 3:**
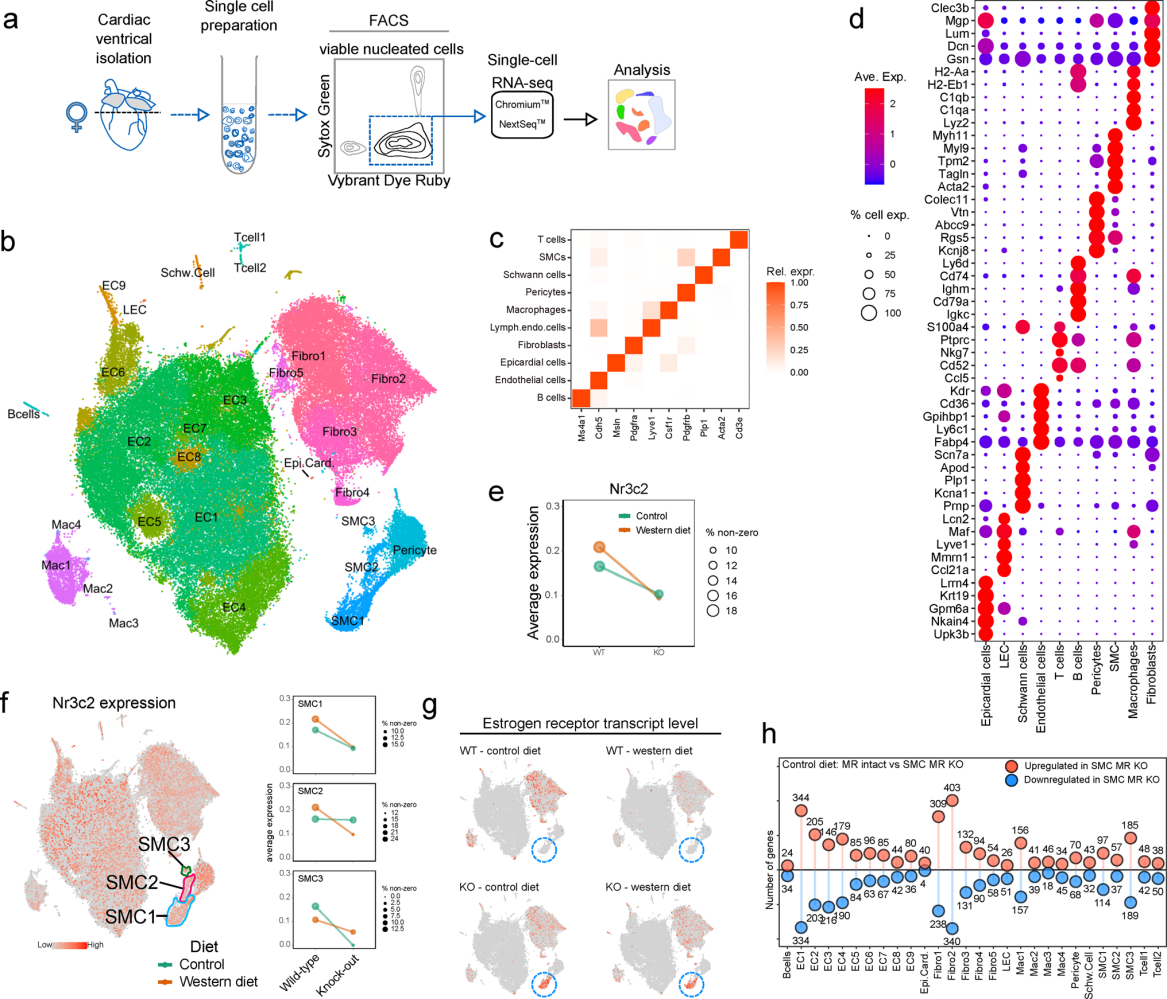
Isolation and analysis of cardiac non-myocyte populations by single-cell RNA sequencing (scRNA-seq). **a** Schematic outlining the experimental procedure for cell isolation and analysis of adult mouse cardiac non-myocytes by scRNA-seq. **b** t-SNE projection of cardiac cell populations identified by scRNA-seq analysis. Each dot represents a cell that is colored based on distinct cell populations. **c** Heatmap showing relative expression of canonical cell type markers for major cell types identified in adult mouse heart. **d** Dot plot for top 5 highly and uniquely expressed genes in each major cardiac cell population identified using an unsupervised analysis. Dot color and size indicate the relative expression and percentage of cells expressing that gene within each cell population, respectively (also see Online Resource 11). **e** Average expression of mineralocorticoid receptor (MR; *Nr3c2*) in coronary smooth muscle cells (SMC) from MR-Intact and SMC-MR knockout mice fed control (Con) and western diet (WD). Dot color and size indicate the diet group and the percentage of cells expressing *Nr3c2* gene within each group, respectively. **f** MR (*Nr3c2*) gene expression (red dots) in cell populations and in 3 SMC clusters identified using an unsupervised analysis. Dot color and size (right plots) indicate the diet group and the percentage of cells expressing *Nr3c2* gene within each group, respectively. **g** Estrogen receptor (*Esr1*) expression (red dots) in cell populations from each treatment group with SMC1 population indicated by blue circle. **h** Lollipop plot summarizing number of up- and downregulated genes (uncorrected *p* < 0.01) in Con-fed SMC-MR-KO mouse heart cells relative to Con-fed MR-Intact cells (also see Online Resource 16)

*SMC-MR deletion alters non-myocyte gene signatures in mice fed control diet.* Since bulk RNA sequencing analysis is largely dominated by cardiomyocyte mRNA, we next performed high-resolution scRNA-seq analysis of the non-myocyte cell populations to elucidate cell-specific changes elicited by WD feeding and potential mechanisms underlying protection via SMC-MR deletion in obesity. Using established protocols (Fig. 3A) [54], our analysis demonstrated a wide diversity of cardiac non-myocyte cell types including multiple clusters of fibroblasts, vascular smooth muscle and endothelial cells, and diverse immune cell types identified by expression of canonical and non-canonical marker genes (Fig. 3B–D, Online Resource 12). The relative proportions of the various cell types did not differ in response to diet or to SMC-MR deletion (Online Resource 13). We confirmed the SMC-specificity of the model with a reduction of *Nr3c2* (the gene for the MR) across all coronary SMC clusters from SMC-MR-KO compared to MR-Intact mice (Fig. 3E and F; Online Resource 14) with no reduction in MR expression in other non-myocyte cell populations in the heart (Online Resource 15). Our first analysis compared gene expression between MR-Intact and SMC-MR-KO mice fed control diet. In SMCs, the most differentially expressed gene in response to SMC-MR deletion was a marked upregulation of the estrogen receptor (*Esr1*) independent of diet feeding (Fig. 3G). We also found significant gene expression differences between genotypes in a cell-specific manner (Fig. 3H, Online Resource 16). Analysis of differentially regulated genes indicates a key feature of SMC-MR-KO is increased expression of major histocompatibility complex (MHC) class 1 genes (*H2-Q7*, *-Q4*, *-Q6*, *H2-Eb1*, and others) in multiple cell types of SMC-MR-KO animals, particularly ECs, macrophages, and fibroblasts corresponding to GO terms associated with immune modulation and T cell-mediated cell targeting (Online Resources 17 and 18). Also noted were increased levels of transcripts corresponding to “response to glucocorticoid” and “response to corticosteroid” in fibroblast and EC subsets, and “angiogenesis” in fibroblasts and SMCs from SMC-MR-KO mice (Online Resource 18). Gene programs corresponding to “angiogenesis” were also frequently downregulated in multiple cell types, in SMC-MR-KO animals, in addition to those involved in “extracellular matrix organization” (in fibroblast subsets) (Online Resources 17 and 18). However, no differences in inflammation, coronary function, or cardiac function were detected relative to control-fed MR-Intact mice (Figs. 1 and 2); hence, the relevance of these differences in gene expression and programs identified in unstressed mice is unclear.

**Fig. 4:**
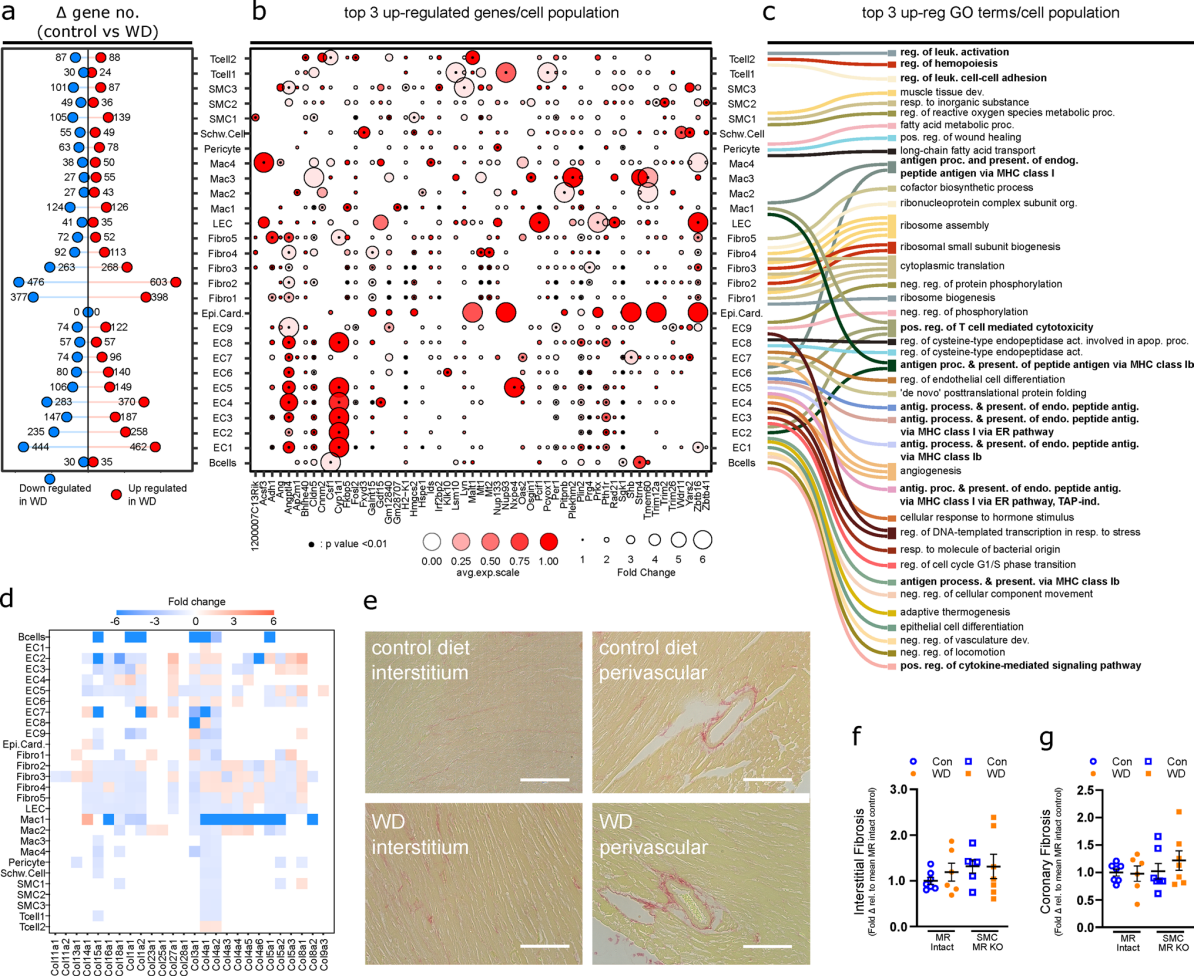
Gene expression changes in cardiac non-myocyte populations in response to western diet (WD) in MR-Intact mice are independent of cardiac fibrosis. **a** Lollipop plot summarizing the number of up- and downregulated genes (uncorrected *p* < 0.01) identified in WD-fed MR-Intact mice relative to control (Con) diet-fed MR-Intact mice (also see Online Resource 19). **b** Dot plot summarizing the relative expression of top 3 upregulated genes in response to WD, in each cardiac cell population. Dot color intensity and size are proportional to the relative gene expression in WD cells and the fold change increment in WD cells compared to the Con cells within each cell population, respectively. Black points at the centers of some dots highlight statistically significant differences in gene expression in WD relative to Con cells (uncorrected *p* < 0.01). **c** Sankey plot summarizing the top 3 statistically significant Gene Ontology (GO) terms (corrected *p* < 0.05) enriched by WD upregulated genes in each cell population. Lines connect GO terms associated with each cell population. Note: not all cell types have 3 statistically significant GO terms (also see Online Resource 22). **d** Heatmap of collagen isoform gene expression in non-myocyte populations from WD versus Con-fed MR-Intact mice (see also Online Resource 24 for all extracellular matrix-related genes). Box color and intensity indicate direction (blue, downregulation; red, upregulation) and magnitude of WD-induced expression changes, respectively. **e** Representative images of cardiac interstitial and perivascular fibrosis assessed by picrosirius red staining. **f**, **g** Levels of interstitial and coronary fibrosis in mice from all groups, relative to levels in hearts of Con-fed MR-Intact mice. Values are mean ± SE with individual data points shown

*scRNA sequencing of non-myocyte cardiac cell populations reveals that WD induces inflammatory pathways in EC and inflammatory cells independent of fibrosis.* We next examined the impact of WD-induced obesity on the cardiac cellulome in MR-Intact mice. This examination of disparate cardiac cell types revealed that WD feeding altered the transcriptional profile of all cell populations examined in MR-Intact mice (Fig. 4A, Online Resource 19). Genes upregulated by WD feeding in MR-Intact mice include genes previously implicated in WD-induced pathology such as *Angptl4* (upregulated in ECs and fibroblasts) [11], *Cyp1a1* (upregulated in ECs) [61], *Plin2* (upregulated in ECs, fibroblasts and macrophages) [41], and *Sgk1* (upregulated in ECs, fibroblasts, pericytes, and B cells) [38] (Fig. 4B). Also upregulated was *Pparγ* (ECs) which positively regulates many of these genes (*Angptl4*, *Plin2*, *Sgk1*) and others (*Fabp4*, *Cd36*, *Tsc22d1*, *Hmox1*, *Aqp7*, *Ucp2*, *Klf4*) that are upregulated in ECs after WD [16] (Online Resource 19). Conversely, genes downregulated by WD feeding in MR-Intact mice include genes involved in energy metabolism (*Ckb*, downregulated in EC, fibroblasts, Schwann and T cells, and SMCs), matrix regulation (*Plod1*, downregulated in EC and macrophages), and clearance of advanced glycosylation end products (*Dcxr*, downregulated in fibroblasts, Schwann cells, and SMCs) (Online Resources 19 and 20).

To determine genetic programs that are up- and downregulated following WD feeding we examined GO terms corresponding to biological processes in differentially expressed gene sets (Online Resources 21 and 22). Among the top biological processes upregulated by WD feeding in MR-Intact mice, we found enrichment of terms associated with regulation/function of immune cells (i.e., antigen processing and presentation, leukocyte activation) (Fig. 4C, Online Resources 21 and 22) and that these were also the most commonly upregulated programs shared across multiple cells populations including ECs and macrophages (Fig. 4C). Further, consistent with exposure to WD, multiple cell populations also activated gene programs linked to fatty acid and lipid metabolism and transport (Online Resource 22). Angiogenesis gene programs were both up- and downregulated by WD feeding (Online Resources 21 and 22) in line with no change in capillary density across groups (Online Resource 23). Lastly, WD feeding did not induce extracellular matrix genes (Online Resource 24), including collagens (Fig. 4D), in non-myocyte populations from MR-Intact mice and GO terms associated with fibrosis (“extracellular matrix organization” and “extracellular structure organization”) were downregulated in major fibroblast clusters (Online Resources 21 and 22). Picrosirius red staining confirmed no change in interstitial or perivascular collagen deposition (i.e., fibrosis) across all treatment groups (Fig. 4E–G; Online Resource 24).

**Fig. 5:**
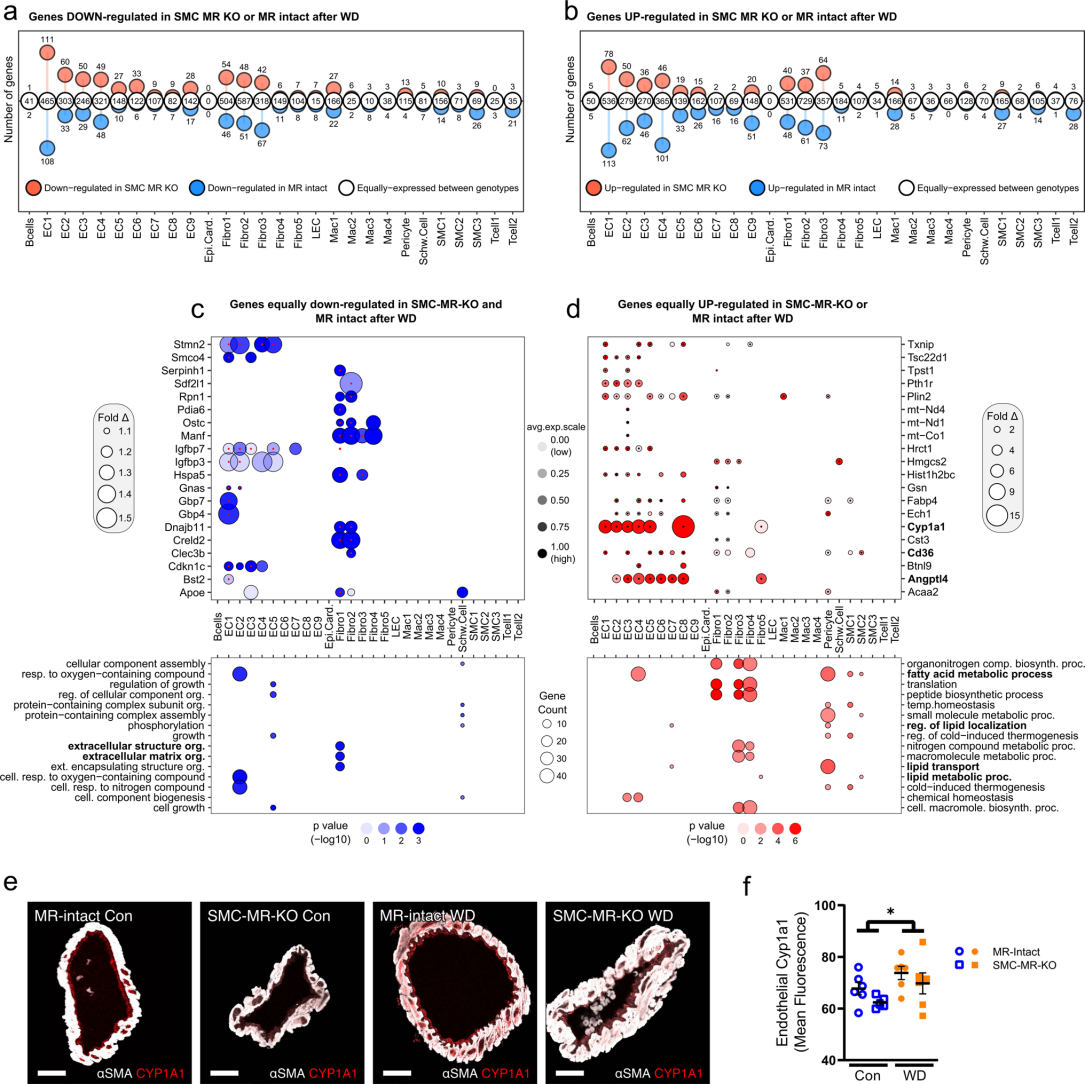
Western diet (WD) feeding induces common and distinct gene activation in MR-Intact and SMC-MR-KO mouse heart cells. **a**, **b** Lollipop plots summarizing genes similarly (white circles) and differentially downregulated (**a**) or upregulated (**b**) by WD feeding in MR-Intact (blue circles) and SMC-MR-KO (red circles) mice by cell population. **c**, **d** Top 20 genes down- or upregulated (**c** and **d**, respectively) following WD in SMC-MR-KO and MR-Intact mice (also see Online Resource 26). Bolded genes indicate those that have been associated with diabetes or obesity. Bottom panels summarize GO terms corresponding to down or upregulated genes (also see Online Resource 27). **e** Representative images and **f** summary data of Cyp1A1 protein expression in coronary endothelium by immunofluorescence. Values are mean ± SE with individual data points shown. *p < 0.05 for noted comparison

*Diabetes- and obesity-associated gene programs are induced by WD feeding independent of MR genotype*. Next, we examined the key common gene expression features induced by WD feeding in MR-Intact and SMC-MR-KO mouse hearts. Indeed, a majority of genes altered by WD were equivalently up- or downregulated across genotypes (Fig. 5A and B; Online Resources 25 and 26). Top downregulated genes included those previously associated with diabetes and obesity, such as *Manf* [66] and *Creld2* [34] that facilitate protein folding (Fig. 5C), and *Igfbp3* and *Igfbp7*, two structurally similar proteins that regulate the bioavailability of IGFs and insulin. As noted for WD-fed MR-Intact mice, WD also downregulated genes associated with extracellular matrix in SMC-MR-KO mice (Fig. 5C, Online Resources 26 and 27) and top upregulated genes also included those implicated in diabetes and obesity, such as *Cd36*, *Txnip*, *Angptl4*, *Cyp1a1*, and *Fabp4* (Fig. 5D). Immunofluorescence confirmed increased protein expression of the top gene upregulated by WD feeding, Cyp1a1, in coronary endothelium independent of genotype (Fig. 5 E and F). Consistent with a change in diet, genes corresponding to programs involved in fatty acid and lipid metabolism were upregulated in both genotypes by WD feeding; however, these were primarily restricted to mural cells (Fig. 5D, Online Resources 26 and 27). Since SMC-MR-KO mice are protected from WD-induced coronary and cardiac dysfunction, these genotype-independent WD-induced gene changes likely represent pathways not involved in this protection.

**Fig. 6:**
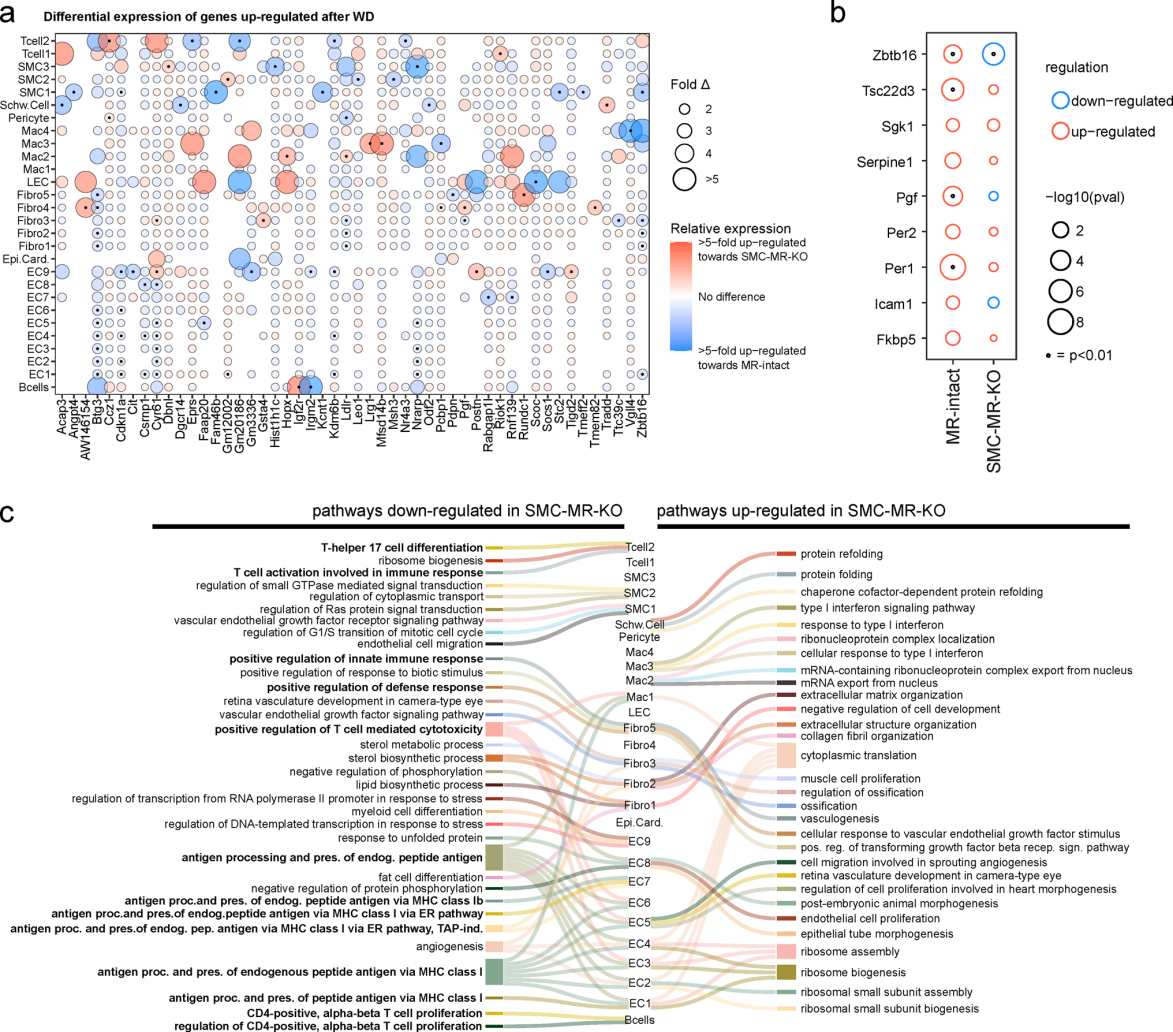
Smooth muscle cell mineralocorticoid receptor knockout (SMC-MR-KO) alters cardiac gene expression response to western diet (WD) feeding. **a** Top 50 genes that are upregulated after WD and differentially expressed between MR-Intact and SMC-MR-KO mouse heart cells. Blue and red circles indicate genes that are more highly expressed in MR-Intact or SMC-MR-KO mouse heart cells, respectively. Black dot (center of some circles) indicates statistical significance of *p* < 0.001 (see Online Resource 28). **b** Changes in expression of MR target genes in cardiac SMCs from MR-Intact and SMC-MR-KO mice fed WD versus Con-fed mice from each genotype. Black dot (center of some circles) indicates statistical significance. **c** Sankey plot summarizing the top 3 statistically significant Gene Ontology (GO) terms (corrected *p* < 0.05) enriched in up- (right) and downregulated (left) genes in WD-fed SMC-MR-KO mice versus WD-fed MR-Intact mice. Lines connect GO terms associated with each cell population. Note: not all cell types have 3 statistically significant GO terms (see Online Resource 29)

*SMC-MR deletion impacts WD-induced gene expression across cell populations, including MR target genes*. Next, we examined genes and gene programs that were more robustly induced or repressed by WD feeding in SMC-MR-KO mice compared to MR-Intact mice. Top genes differentially induced or suppressed between genotypes after WD generally followed a cell-specific pattern (Fig. 6, Online Resource 28). Notably, genes whose expression was increased more robustly in MR-Intact mouse hearts included the anti-proliferation genes *Btg3* (ECs and fibroblasts) and *Cdkn1a* (ECs and SMC1), as well as angiogenic genes *Cyr61* (ECs) and *Nrarp* (ECs), and the cholesterol uptake regulator *Ldlr* (fibroblasts and pericytes). Further, genes more robustly expressed in MR-Intact mouse hearts after WD feeding included MR- and GR-sensitive genes such as *Zbtb16*, *Fam46b*, and *Angptl4* in SMC populations [10]. Closer examination of reported MR-sensitive genes showed upregulation of a number of these genes in SMCs from MR-Intact, but not SMC-MR-KO, mouse hearts after WD (Fig. 6B). Indeed, *Zbtb16*, a key transcriptional repressor, and *Pgf*, a pro-angiogenic and pro-inflammatory MR target gene, were upregulated by WD in MR-Intact SMCs and downregulated or not changed, respectively, in SMC-MR-KO SMCs after WD.

*SMC-MR deletion attenuates WD-induced inflammation across the cardiac cellulome.* The most significant differences in gene expression programs activated by WD in the two genotypes were those related to immune activity. Direct comparison of genetic programs among genes down- and upregulated in SMC-MR-KO mice by WD, versus WD-fed MR-Intact mice, further revealed reduced WD-induced immune response in these mice (Fig. 6C; Online Resource 29). Specifically, downregulated GO terms in WD-fed SMC-MR-KO hearts were enriched for terms associated with antigen processing and presentation (ECs and Mac1) as well as positive regulation of immune responses (ECs, Mac1, Fibro5). Intriguingly, in control diet-fed mice, many of these genes and GO terms were more highly expressed in SMC-MR-KO heart ECs, compared to MR-Intact heart ECs (Online Resources 16 and 18). Further, downregulation of GO terms related specifically to T cell immunity were enriched in B cells (*‘*CD4-positive, alpha–beta T cell proliferation’), ECs and Mac1 (‘positive regulation of T cell mediated cytotoxicity’), and T cell2 (‘T cell activation’ and ‘differentiation’) (Fig. 6C; Online Resource 29). The enrichment of genes related to leukocyte trafficking and inflammation among the most statistically significant programs in multiple cell types (Fig. 6C) identifies a key distinction in the phenotypes of MR-Intact and SMC-MR-KO mouse hearts after WD.

**Fig. 7:**
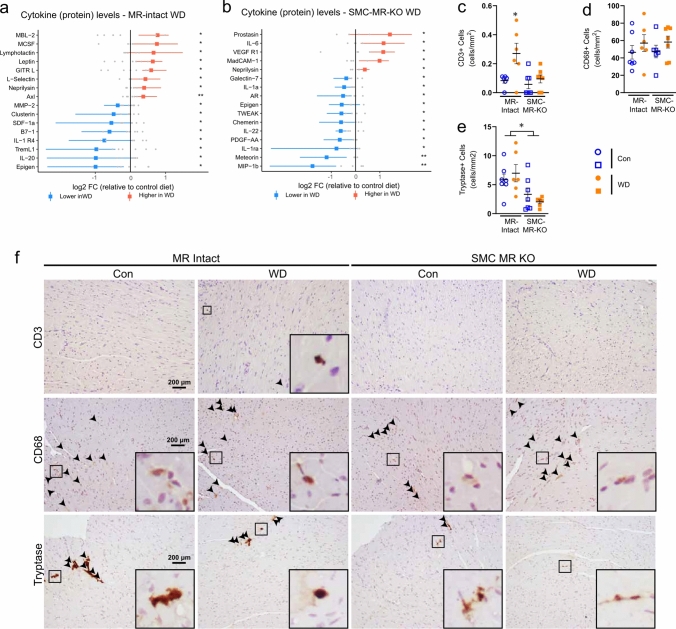
Smooth muscle cell mineralocorticoid receptor knockout prevents obesity-associated cardiac inflammation. Differentially expressed cardiac cytokines in **a** WD-fed MR-Intact mice and **b** WD-fed SMC-MR-KO mice versus Con-fed mice of each genotype. Red, upregulation; blue, downregulation; colored dot is mean with bar indicating spread of data in WD group; gray dots indicate individual data points in Con group. *p < 0.05; **p < 0.01. Cardiac **c** CD3+ , **d** CD68+ , and **e** Tryptase+ cells assessed by immunohistochemistry and **f** representative images by group, arrows point to positively stained cells, insets include zoomed in view of positive staining. Values are mean ± SE with individual data points shown. *p < 0.05 versus all other groups or noted comparison

Next, using orthogonal approaches, we sought to validate differences in inflammatory responses in the two genotypes identified by scRNA-seq. First, a cytokine array confirmed a pro-inflammatory cytokine signature in hearts from WD-fed MR-Intact mice. Notably, WD feeding upregulated the T cell chemokine lymphotactin and the adhesion molecule L-selectin (Fig. 7A). In addition, factors implicated in promotion of local immune/cytokine signaling including M-CSF, GITR-L, and Axl were upregulated by WD feeding in MR-Intact hearts (Fig. 7A). Deletion of SMC-MR altered the cardiac cytokine profile in both control (Online Resource 30) and WD-fed SMC-MR-KO mice (Fig. 7B, Online Resource 30). In contrast to WD-fed MR-Intact mice, SMC-MR-KO mice fed a WD exhibited cytokine changes indicative of reduced cardiac inflammation compared to control-fed SMC-MR-KO mice. Specifically, the pro-inflammatory mediators TWEAK, PDGF-AA, MIP-1b, IL-22, and IL-1a were reduced in WD-fed SMC-MR-KO mice (Fig. 7B). Accordingly, the impact of SMC-MR deletion on WD-induced cardiac immunity was further explored by immunohistochemistry and flow cytometry. In MR-Intact mice, WD feeding increased cardiac CD3+ T cells but not CD68+ monocytes/macrophages (Fig. 7C–F, Online Resource 31). Single-cell transcriptomics revealed upregulation of macrophage activation markers *Cd86* and *Cd83* (Mac1; Online Resource 19) in WD-fed MR-Intact mice consistent with a trend for increased cardiac CD11b + CD80+ macrophages by flow cytometry (*p* = 0.07; Online Resource 31). These changes in cardiac leukocytes were prevented in WD-fed SMC-MR-KO mice. Furthermore, consistent with downregulation of the ‘Th17 cell differentiation’ GO term in the Tcell2 cluster from WD-fed SMC-MR-KO mice compared to WD-fed MR-Intact mice (Fig. 6C), SMC-MR deletion was associated with reduced cardiac Th17 cells (Online Resource 31). Lastly, SMC-MR-KO mice exhibited a reduction in cardiac mast cells, a cell population recently implicated in diabetes-associated cardiac dysfunction [25], independent of diet feeding (Fig. 7E and F, Online Resource 31). Together, these data implicate SMC-MR signaling as necessary for development of coronary and cardiac dysfunction in obesity and as a critical driver of obesity-associated cardiac inflammation, not only through changes in SMC but across the cardiac cellulome, supporting a role for SMC-MR in intercellular crosstalk underlying coronary and cardiac dysfunction in obesity.

## Discussion

Our data demonstrate critical involvement of SMC-MR in obesity-associated coronary and cardiac dysfunction in female mice. Specifically, in obese female mice, SMC-MR deletion prevented the decline in coronary endothelium-dependent vasodilation and increase of vasoconstriction as well as impaired cardiac diastolic function, but not cardiac hypertrophy. Importantly, these benefits of SMC-MR deletion occurred independent of changes in blood pressure, aortic stiffening, and obesity-associated adipose inflammation, metabolic dysregulation, and kidney injury. Further, scRNA-seq revealed a distinct inflammatory gene profile in non-myocyte populations from obese mice that was independent of cardiac fibrosis and associated with cardiac leukocyte infiltration. This obesity-associated cardiac inflammatory phenotype was generally prevented by SMC-MR deletion. Together, these data provide unique insight and support the emerging paradigm of a vascular origin of cardiac dysfunction in obese females [46, 50], patients at high risk for developing HFpEF [27, 58].

Involvement of MR signaling in obesity-associated cardiovascular morbidity and mortality is supported by both clinical and preclinical work, from us [4, 6, 7, 13, 21] and others [19, 33, 53], utilizing MR antagonists. In obese rodent and swine models, MR blockade with spironolactone or eplerenone mitigated endothelial dysfunction and vascular remodeling as well as diastolic dysfunction, cardiac oxidative stress, fibrosis, and inflammation [4, 6, 7, 13, 21, 53]. Mechanistically, several prior studies have revealed an important role for EC MR signaling underlying vascular and cardiac dysfunction in obesity. Specifically, deletion of EC MR prevented obesity-associated endothelial dysfunction in aorta and mesenteric arterioles as well as cardiac diastolic dysfunction [12, 31, 32]. Improved vascular function in obesity following EC MR deletion was associated with modulation of reactive oxygen species production/degradation and NO bioavailability [12, 31], while prevention of cardiac dysfunction was associated with reduced cardiac pro-oxidant and pro-inflammatory signaling [32]. Improved cardiac function in obese female mice with EC MR deletion may also be due, in part, to attenuated obesity-associated aortic stiffening in these mice [31]. Our data expand this previous work by demonstrating, for the first time, a critical role of SMC-MR signaling underlying coronary and cardiac diastolic dysfunction in obese females. That obesity-associated endothelial dysfunction was prevented by SMC-MR deletion supports recent evidence that SMC dysfunction may precede [22], and contribute to development of, impaired endothelium-dependent vasodilation in obesity. Taken together, these studies highlight novel, independent roles of MR signaling in vascular cells in the pathogenesis of obesity-associated cardiac dysfunction in females. Importantly, the protection afforded by SMC-MR deletion occurs independent of changes in traditional risk factors (blood pressure, glucose intolerance, hypercholesterolemia, kidney injury) and aortic stiffening suggesting local SMC-MR-dependent mechanisms of dysfunction within the cardiac microenvironment.

Systemic and cardiac inflammation have been implicated as requisite contributors in the etiology of obesity-associated cardiac impairments, including HFpEF [18, 50, 60]. Indeed, cardiac inflammation has been suggested to precede and contribute to other common characteristics of cardiac diastolic dysfunction, such as fibrosis and sarcomeric stiffening, in the setting of co-morbid conditions [50, 63]. Consistent with this paradigm, in our hands, WD-fed MR-Intact female mice do not exhibit cardiac fibrosis despite increased cardiac expression of pro-inflammatory cytokines (e.g., neprilysin, M-CSF). These data suggest that WD-induced diastolic impairments in this model likely involve sarcomeric stiffening (*i.e.*, titin hypophosphorylation) [28] and/or altered cardiomyocyte calcium handling [44, 62].

Unbiased scRNA-seq data confirmed upregulation of inflammatory pathways across cardiac non-myocyte populations by WD feeding independent of a pronounced upregulation of extracellular matrix-related genes. These data further highlight a vascular contribution to cardiac inflammation in obesity via upregulation of gene programs for EC antigen presentation and processing via MHC class I molecules in conjunction with upregulation of genes associated with T cell-mediated cytotoxicity in cardiac ECs and macrophages. These gene changes correspond to cardiac infiltration of CD3+ T cells supporting recent evidence that increased endothelial MHC class I molecule expression enhances T cell transmigration [39]. Further, cytokine analysis reveals obesity-associated upregulation of the adhesion molecule L-selectin, the T cell chemokine lymphotactin, and the T cell costimulatory ligand GITR-L. Interestingly, GITR-L engagement of T cell GITR has been reported to reduce susceptibility of effector T cells to suppression by T regulatory cells [55]. Prior work has established a link between cardiac T cell infiltration and the development of systolic dysfunction in response to cardiac pressure overload [48]. Our data extend these findings and, to our knowledge, are the first to suggest a role for cardiac T cell infiltration in the inflammatory processes contributing to cardiac diastolic dysfunction in obese female mice.

MR-dependent inflammation has been implicated in a variety of disease states, including obesity. Indeed, global MR blockade reduces obesity-associated adipose [4, 7, 26], cardiac [4, 6, 7, 26], and vascular [4, 13] inflammation. Our data delineate a novel role for SMC-MR signaling as a primary contributor to coronary and cardiac, but not adipose, inflammation in obesity. Specifically, obesity-associated upregulation of aortic adhesion molecules and endothelial MHC class I molecules as well as cardiac T cell infiltration were prevented in obese SMC-MR knockout mice. This protective effect of SMC-MR deletion corresponded with generally anti-inflammatory shifts in cardiac cytokines in obese, versus lean, SMC-MR knockout mice and prevention of obesity-associated upregulation of the pro-inflammatory SMC-MR target *Pgf*. Similar prevention of cardiac inflammation by SMC-MR deletion, including prevention of cardiac *Pgf* upregulation, was recently reported in male mice subjected to cardiac pressure overload [35]. Further, cardiac leukocyte infiltration in response to pressure overload consisted entirely of CD3-CD11b+ myeloid cells and was prevented by SMC-MR deletion [35]. In conjunction with the present results, these data argue for significant context-specificity of SMC-MR-dependent mechanisms of cardiac inflammation and leukocyte recruitment. Intriguingly, our data reveal a novel reduction of cardiac mast cells in SMC-MR-KO mice independent of diet treatment. Recent evidence in female *db*/*db* mice demonstrated a critical role for activation/degranulation of resident cardiac mast cells in diabetes-associated cardiac leukocyte infiltration and diastolic dysfunction [25]. Thus, reduced cardiac mast cells may be a unifying mechanism of cardioprotection accounting for reduced cardiac dysfunction in SMC-MR-KO mice following obesity, coronary ligation [24], aging [14, 36], and pressure overload [35]. Our use of inducible SMC-MR deletion suggests that any impact of SMC-MR signaling on cardiac mast cells is not of developmental origin and potential SMC-MR-dependent mechanisms of cardiac mast cell recruitment/maturation are warranted.

Mechanistically, our data suggest that enhanced SMC estrogen signaling in SMC-MR-KO mice may contribute to prevention of obesity-associated cardiovascular dysfunction. Indeed, SMC-MR deletion resulted in pronounced upregulation of SMC estrogen receptor (ER; *Esr1*) gene expression. This finding is consistent with recent reports of ER upregulation in macrophages/Kupffer cells [69] and EC [5] following cell-specific MR deletion. In the latter study, double deletion of EC MR and ER eliminated the prevention of obesity-associated endothelial dysfunction afforded by EC MR deletion alone [5]. While potential SMC ER-dependent mechanisms of cardioprotection remain unclear in the present study, recent evidence supports paracrine SMC ER signaling as a contributor to endothelial healing/regeneration following vascular injury [51, 68]. Since this study was performed only in female mice, whether SMC ER upregulation might occur in male SMC-MR-KO mice is not known. We focused on female mice in this study in light of the high prevalence of coronary microvascular dysfunction in women with co-morbid conditions and its close association with cardiac dysfunction [27, 49, 58].

Our study also provides a useful resource for examining pathways that may be important for cardiac remodeling, but not WD-induced diastolic dysfunction. These include genes such as such *Angptl4*, *Cd36*, *Cyp1a1* and *Pparγ* and others which are upregulated after WD in both MR-Intact and SMC-MR-KO mouse hearts. Examination of these pathways to abrogate cardiovascular remodeling have been active areas of research for many years and master regulators, such as *Pparγ*, may be key for WD-induced changes in EC phenotypes we have determined here by scRNAs-seq. Further, our proteomic analysis also detected neprilysin—an enzyme that degrades natriuretic peptide and angiotensin II—at higher levels after WD in both genotypes. Indeed, targeting neprilysin while inhibiting the angiotensin receptor improves morbidity and mortality associated with heart failure [42]. However, modulating the activity of these elements that are activated in both MR-Intact and SMC-MR-KO mouse hearts may have limited effect on diastolic impairment of heart function in obesity.

In summary, this study reveals a central role for SMC-MR signaling in the development of obesity-associated coronary and cardiac diastolic dysfunction in female mice. This is the first report, to our knowledge, of obesity-associated transcriptomic changes across the cardiac non-myocyte cellulome. This unbiased approach revealed cardiac inflammation, associated with lymphocyte infiltration, and hypertrophy independent of fibrosis in obese female hearts. SMC-MR deletion mitigated obesity-associated cardiac and coronary inflammation and dysfunction, but not hypertrophy, potentially involving reduced cardiac mast cells and enhanced SMC estrogen signaling that warrant further investigation. These results shed new light on vascular mechanisms of obesity-associated cardiac dysfunction in premenopausal women and provide rationale for further study of MR inhibition and pathways downstream of SMC-MR as sex-specific strategies to treat cardiac and coronary dysfunction, critical contributors to development of HFpEF in obese women.

### Supplementary Information

Below is the link to the electronic supplementary material.

Supplementary file1 (PDF 352 KB)

Supplementary file2 (XLSX 60 KB)

Supplementary file3 (PDF 618 KB)

Supplementary file4 (XLSX 708 KB)

Supplementary file5 (PDF 465 KB)

Supplementary file6 (XLSX 548 KB)

Supplementary file7 (PDF 301 KB)

Supplementary file8 (XLSX 106 KB)

Supplementary file9 (XLSX 684 KB)

Supplementary file10 (PDF 524 KB)

Supplementary file11 (XLSX 137 KB)

Supplementary file12 (PDF 929 KB)

Supplementary file13 (XLSX 129 KB)

Supplementary file14 (XLSX 38 KB)

Supplementary file15 (XLSX 11446 KB)

Supplementary file16 (XLSX 102 KB)

Supplementary file17 (PDF 283 KB)

## Data Availability

The datasets generated and/or analyses performed in the present study are available from the corresponding author on reasonable request.
