# Supplementary material for: Multi-omic analysis of the cardiac cellulome defines a vascular contribution to cardiac diastolic dysfunction in obese female mice
Source: Basic Res Cardiol. 2023 Mar 29;118(1):11. doi: 10.1007/s00395-023-00983-6 (PMC10060343; doi:10.1007/s00395-023-00983-6)
Supplement: Supplementary file 1 — Supplementary file1 (PDF 352 KB) [file 395_2023_983_MOESM1_ESM.pdf]

SUPPLEMENTAL MATERIAL

Multi-omic analysis of the cardiac cellulome defines a vascular contribution to cardiac diastolic dysfunction in obese female mice

Malathi S. I. Dona[1#], Ian Hsu[1#], Alex I. Meuth[2,4], Scott M. Brown[2,4], Chastidy Bailey[2,4], Christian G. Aragonez[2,4], Jacob J. Russell[2,4], Crisdion Krstevski[1], Annayya R. Aroor[4,5], Bysani Chandrasekar[3-5], Luis A. Martinez-Lemus[3,6], Vincent G. DeMarco[4,5], Laurel A. Grisanti[2], Iris Z. Jaffe[7], Alexander R. Pinto[1,8*], Shawn B. Bender[2-4*]

[1]Baker Heart and Diabetes Research Institute, Melbourne, Victoria, Australia

[2]Biomedical Sciences and [3]Dalton Cardiovascular Research Center; University of Missouri, Columbia, Missouri, USA

[4]Research Service, Harry S Truman Memorial Veterans Hospital, Columbia, Missouri, USA

[5]Medicine and [6]Medical Pharmacology & Physiology, University of Missouri School of Medicine, Columbia, Missouri, USA

[7]Molecular Cardiology Research Institute, Tufts Medical Center, Boston, Massachusetts, USA

[8]Centre for Cardiovascular Biology and Disease Research, La Trobe University, Melbourne, Australia
[#]Equal contribution

Running title: Vascular role in cardiac dysfunction in obesity

***Correspondence:**

Shawn B. Bender, Ph.D.
Harry S. Truman Memorial Veterans' Hospital and University of Missouri
Biomedical Sciences
E102 Vet Med Bldg
Columbia, MO 65211
Phone:573-814-6000 x53731
Fax:573-884-6890
Email: benders@missouri.edu

Alexander R. Pinto, Ph.D.
Baker Heart and Diabetes Institute
75 Commercial Rd
Prahran, Victoria
Australia 3004
Phone: +61 8532 1111
Email: alex.pinto@baker.edu.au

Online Resource 1. Primer sequences for real-time quantitative PCR

Gene Name	Primer Sequence (5' → 3')	
	**Forward**	**Reverse**
**ITGAX (CD11c)**	CTGGATAGCCTTTCTTCTGCTG	GCACACTGTGTCCGAACTC
**ADGRE1 (F4/80)**	CTTTGGCTATGGGCTTCCAGTC	GCAAGGAGGACAGAGTTTATCGTG
**GAPDH**	TCACCACCATGGAGAAGGC	GCGAAGCAGTTGGTGGTGCA
**ICAM-1**	AACCGCCAGAGAAAGATCAG	TGTGACAGCCAGAGGAAGTG
**PECAM-1**	GAGCCCAATCACGTTTCAGTTT	TCCTTCCTGCTTCTTGCTAGCT
**TNF-α**	GCCTCTTCTCATTCCTGCTTG	CTGATGAGAGGGAGGCCATT
**VCAM-1**	CTTCATCCCCACCATTGAAG	TGAGCAGGTCAGGTTCACAG

Online Resource 2. Phenotypic characteristics of study animals by group

	**Control**	**Western**	**Western + Spiro**
Body Weight (g)	22.0 ± 0.7	25.7 ± 1.0*	25.0 ± 0.9*
Periovarian Fat Weight (mg)	431 ± 50	834 ± 186*	790 ± 113*

Values are mean ± SE, n=7/group; *p<0.05 vs Control.

Online Resource 3. Phenotypic characteristics of study animals by group

	MR-Intact Control	MR-Intact Western	SMC MR KO Control	SMC MR KO Western
Body Weight (g)	22.3 ± 0.5	29.9 ± 1.0*	21.5 ± 0.3	26.8 ± 1.0*†
Heart Weight (mg)	114 ± 3	123 ± 4*	113 ± 2	121 ± 3*
HW/TL	64 ± 2	69 ± 2*	63 ± 1	67 ± 1*
Periovarian Fat Weight (mg)	740 ± 82	2104 ± 147*	676 ± 57	1540 ± 153*†
Systolic Blood Pressure (mmHg)	120 ± 7	119 ± 7	117 ± 8	124 ± 7
Pulse Wave Velocity (cm/s)	3.8 ± 0.4	3.8 ± 0.3	3.5 ± 0.2	3.9 ± 0.2
Plasma Parameters				
Blood Glucose (mg/dl)	156 ± 11	183 ± 10*	137 ± 7	191 ± 8*
Plasma Insulin (pg/ml)	172 ± 25	266 ± 50**	116 ± 24	319 ± 35*
Plasma Cholesterol (mg/dl)	75 ± 4	115 ± 7*	70 ± 5	110 ± 7*
Plasma Triglycerides (mg/dl)	53 ± 6	56 ± 2	47 ± 5	51 ± 9
Plasma Potassium (mEq/ml)	4.0 ± 0.2	3.8 ± 0.1	4.0 ± 0.2	4.0 ± 0.2
Plasma Aldosterone (pg/ml)	763 ± 70	1159 ± 160*	800 ± 61	1315 ± 152*
Urine Parameters				
Proteinuria (mg/mg creatinine)	1.3 ± 0.2	2.2 ± 0.2*	1.1 ± 0.1	2.3 ± 0.3*
BUN (mg/dl)	2916 ± 398.2	4184 ± 538*	2149 ± 117	4222 ± 422*

Values are mean ± SE, n=5-23; *p<0.05 vs genotype control, **p=0.08 vs genotype control, †p<0.05 vs MR-intact Western

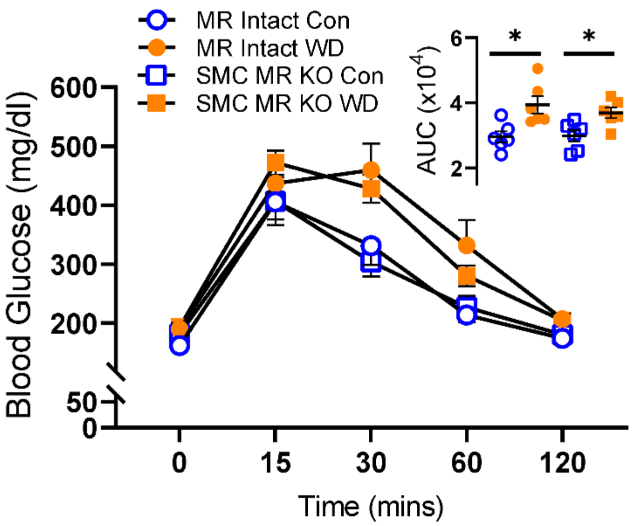

Online Resource 4. *Smooth muscle cell mineralocorticoid receptor knockout (SMC-MR-KO) does not prevent western diet (WD)-induced glucose intolerance in female mice.* Glucose excursions over time during a glucose tolerance test (ip, 1 g/kg) and glucose area under the curve (AUC; inset) in MR-intact and SMC-MR-KO mice fed control (Con) and WD for 16 weeks. Values are mean±SE with individual data points shown (inset), n=6/group, *p<0.05 for indicated comparison.

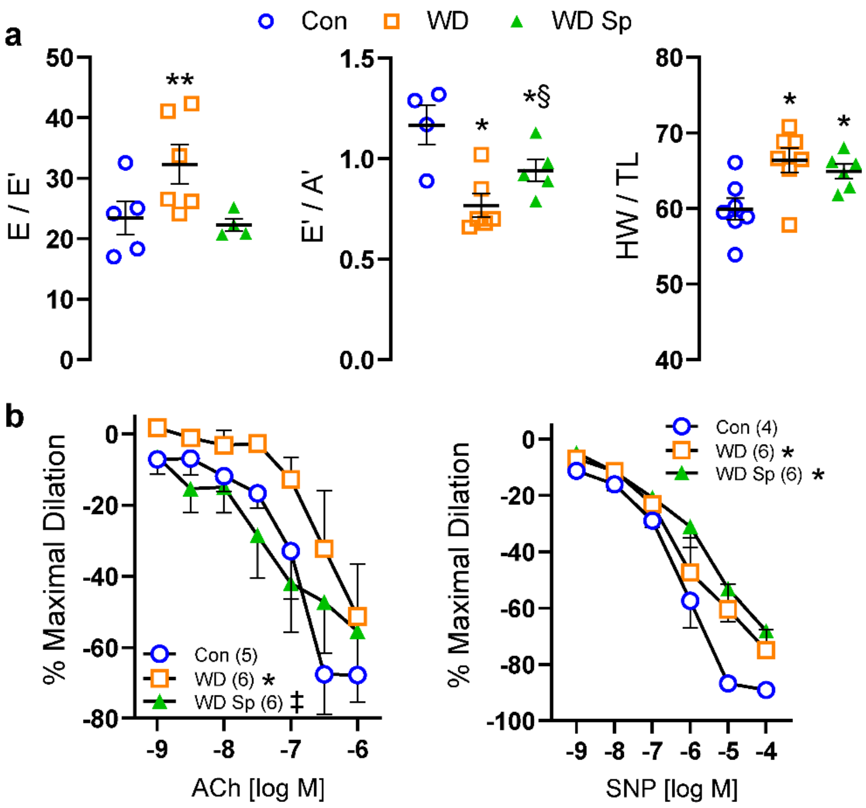

Online Resource 5. *Systemic mineralocorticoid blockade with spironolactone (Sp) prevents western diet (WD)-induced cardiac and coronary vascular dysfunction in female mice.* (**A**) Indices of cardiac diastolic function, specifically estimated left ventricular filling pressure (E/E') and early-to-late diastolic septal annulus motion ratio (E'/A'), and cardiac weights (heart weight-to-tibia length ratio; HW/TL) in Con and WD fed mice. (**B**) Vasodilator responses of isolated coronary arteries to endothelium-dependent (acetylcholine, ACh) and -independent (sodium nitroprusside, SNP) agonists. Values are mean±SE with individual data points shown (A) and sample size indicated in parentheses (B); *p<0.05 versus Con, **p<0.05 versus all other groups, ‡p<0.05 versus WD ,§p=0.08 versus WD.

Online Resource 6. Cardiac function outcomes by experimental group

Parameter	**Control**	**Western**	**Western + Spiro**
Heart Rate (bpm)	542 ± 32	512 ± 27	525 ± 9
Morphological parameters			
SWTd (mm)	0.82 ± 0.03	0.89 ± 0.09	0.86 ± 0.04
PWTd (mm)	0.71 ± 0.03	0.81 ± 0.03§	0.73 ± 0.05
LVIDd (mm)	3.58 ± 0.07	3.98 ± 0.13*	3.55 ± 0.12†
LVIDs (mm)	1.98 ± 0.16	2.38 ± 0.16	2.01 ± 0.16
RWT	0.43 ± 0.01	0.43 ± 0.04	0.45 ± 0.02
LA (mm)	1.65 ± 0.09	2.02 ± 0.05*	1.97 ± 0.12*
Ao (mm)	1.40 ± 0.04	1.49 ± 0.09	1.48 ± 0.04
LA/Ao	1.18 ± 0.06	1.39 ± 0.10§	1.33 ± 0.07
Diastolic parameters			
E ($m \cdot s^{-1}$)	531.4 ± 28.7	602.3 ± 14.8	639.2 ± 33.8
E' ($m \cdot s^{-1}$)	23.7 ± 2.5	19.6 ± 1.9	26.4 ± 2.9
A' ($m \cdot s^{-1}$)	21.5 ± 2.0	25.5 ± 1.9	27.8 ± 2.0
E/E'	23.4 ± 2.8	32.3 ± 3.3*	22.2 ± 1.0†
E'/A'	1.17 ± 0.10	0.77 ± 0.06*	0.94 ± 0.06*‡
IVRT (ms)	12.7 ± 0.9	12.7 ± 0.9	13.0 ± 0.9
Systolic parameters			
EF (%)	75 ± 4	71 ± 3	76 ± 3
FS (%)	44 ± 3	40 ± 2	45 ± 3

SWTd, septal wall thickness-diastole; PWTd, posterior wall thickness-diastole; RWT, relative wall thickness; LVIDd, LV inner dimension-diastole; LVIDs, LV inner dimension-systole; EF, ejection fraction; FS, fractional shortening; LA, left atrial diameter; Ao, aortic diameter; A′, peak late septal annular velocity; E′, early peak septal annular velocity; IVRT, isovolumic relaxation time; E, velocity of early mitral inflow; E/E' index of LA filling pressure; MPI, myocardial performance index. Values are mean ± SE, N=4-6. *p<0.05 versus Control; †p<0.05 versus Western Diet; ‡p=0.08 versus Western Diet; §p=0.07 versus Control.

Online Resource 7. Cardiac function outcomes by experimental group

Parameter	**MR-Intact Control**	**MR-Intact Western**	**SMC MR KO Control**	**SMC MR KO Western**
Heart Rate (bpm)	423 ± 12	424 ± 14	406 ± 18	434 ± 16
Morphological parameters				
SWTd (mm)	0.77 ± 0.03	0.91 ± 0.03*	0.70 ± 0.03	0.81 ± 0.03‡
PWTd (mm)	0.68 ± 0.02	0.77 ± 0.03*	0.70 ± 0.04	0.74 ± 0.03
LVIDd (mm)	3.51 ± 0.09	3.47 ± 0.08	3.49 ± 0.10	3.49 ± 0.08
LVIDs (mm)	2.37 ± 0.12	2.23 ± 0.07	2.41 ± 0.10	2.30 ± 0.10
RWT	0.42 ± 0.02	0.49 ± 0.01*	0.40 ± 0.02	0.45 ± 0.02‡
LA (mm)	1.59 ± 0.06	1.74 ± 0.09	1.40 ± 0.07	1.53 ± 0.06†
Ao (mm)	1.43 ± 0.09	1.32 ± 0.03	1.35 ± 0.09	1.31 ± 0.03
LA/Ao	1.14 ± 0.08	1.33 ± 0.07*	1.05 ± 0.05	1.18 ± 0.04†
Diastolic parameters				
E ($m \cdot s^{-1}$)	477.1 ± 18.2	551.9 ± 16.9*	539.2 ± 17.4	571.6 ± 22.9
E' ($m \cdot s^{-1}$)	19.5 ± 0.8	18.5 ± 1.4	21.0 ± 1.4	23.1 ± 1.9†
A' ($m \cdot s^{-1}$)	16.9 ± 0.8	19.9 ± 1.1§	19.0 ± 0.9	20.1 ± 1.3
E/E'	25.2 ± 0.8	30.6 ± 1.9*	26.3 ± 1.5	24.9 ± 1.6†
E'/A'	1.20 ± 0.04	0.86 ± 0.05*	1.12 ± 0.07	1.17 ± 0.08†
IVRT (ms)	15.4 ± 0.7	16.8 ± 1.0	15.1 ± 1.1	14.2 ± 0.8
Systolic parameters				
EF (%)	61 ± 2	66 ± 2§	60 ± 2	62 ± 2
FS (%)	33 ± 2	35 ± 2	31 ± 1	33 ± 1

SWTd, septal wall thickness-diastole; PWTd, posterior wall thickness-diastole; RWT, relative wall thickness; LVIDd, LV inner dimension-diastole; LVIDs, LV inner dimension-systole; EF, ejection fraction; FS, fractional shortening; LA, left atrial diameter; Ao, aortic diameter; A′, peak late septal annular velocity; E′, early peak septal annular velocity; IVRT, isovolumic relaxation time; E, velocity of early mitral inflow; E/E' index of LA filling pressure; MPI, myocardial performance index. Values are mean ± SE, N=9-14. *p<0.05 versus MR-Intact Control; †p<0.05 versus MR-Intact Western; ‡p<0.05 versus SMC MR KO Control; §p=0.07 versus MR-Intact Control.

Online Resource 8. Diameters of isolated coronary vessels by experimental group

	**Control**	**Western**	**Western + Spiro**	
Diameter (μm)	243 ± 4	257 ± 9	256 ± 6	
	**MR-Intact Control**	**MR-Intact Western**	**SMC MR KO Control**	**SMC MR KO Western**
Diameter (μm)	201 ± 10	226 ± 8	214 ± 7	212 ± 8

Values are mean ± SE, n=5-14; Diameter calculated from circumference following the normalization procedure.

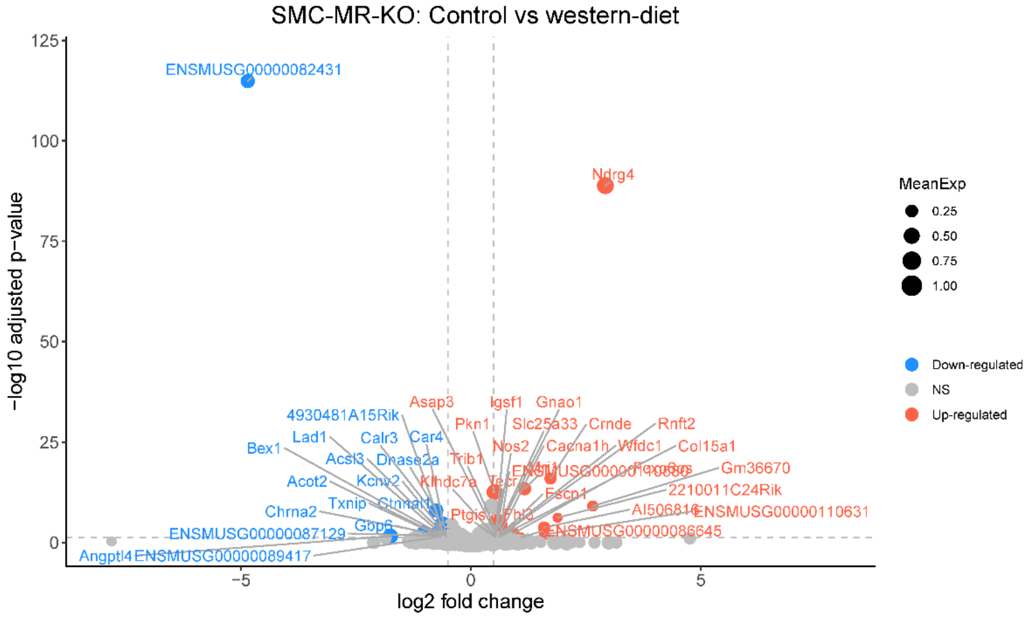

Online Resource 9. *Western diet (WD) feeding altered cardiac gene expression in smooth muscle cell mineralocorticoid receptor knockout (SMC-MR-KO) mice.* Analysis of cardiac transcripts (13,565 transcripts) revealed differential expression (log2 fold change >0.5, corrected p<0.05) of 43 transcripts induced by WD feeding in SMC-MR-KO mice (17 downregulated, blue dots; 26 upregulated, red dots).
