# Supplementary material for: Multi-omic analysis of the cardiac cellulome defines a vascular contribution to cardiac diastolic dysfunction in obese female mice
Source: Basic Res Cardiol. 2023 Mar 29;118(1):11. doi: 10.1007/s00395-023-00983-6 (PMC10060343; doi:10.1007/s00395-023-00983-6)
Supplement: Supplementary file 3 — Supplementary file3 (PDF 618 KB) [file 395_2023_983_MOESM3_ESM.pdf]

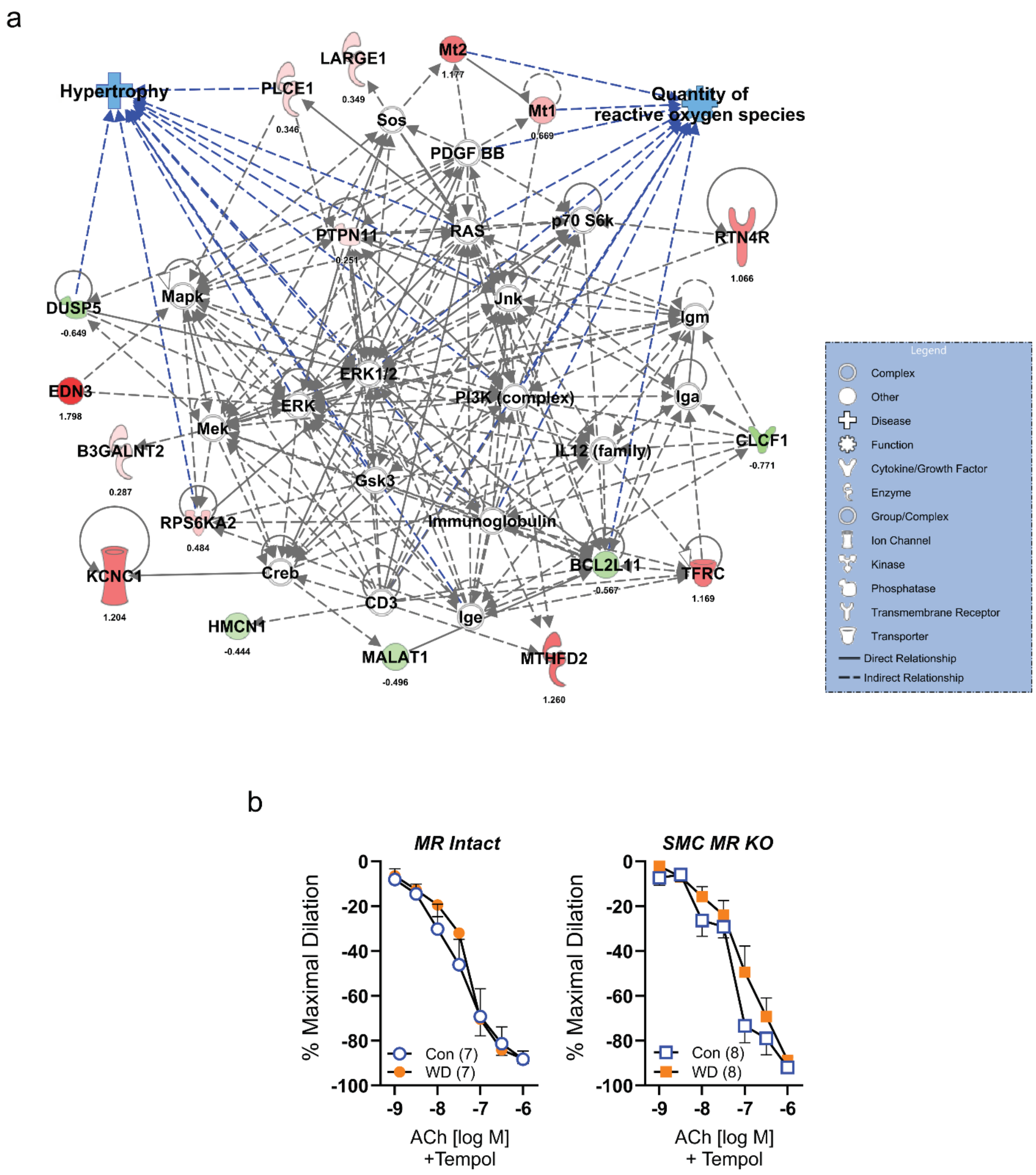

Online Resource 11. *Western diet (WD) feeding alters the cardiac transcriptome in MR-intact mice and induces coronary oxidative stress in female mice that is prevented by smooth muscle cell mineralocorticoid receptor knockout (SMC-MR-KO).* (**a**) Top differentially regulated IPA network (IPA score=39; green nodes, downregulated; red nodes, upregulated) in WD-fed MR-intact mice versus control (Con)-fed MR-intact mice with log2 fold changes and enriched biological processes indicated. (**b**) Vasodilator responses of isolated coronary arteries to the endothelium-dependent vasodilator acetylcholine (ACh) in the presence of the superoxide dismutase mimetic Tempol (1 mM). Compare to Figure 1C to view impact of Tempol treatment across groups, especially in vessels from WD-fed MR-intact mice. Values are mean±SE, sample size in parentheses.
