# Supplementary material for: Multi-omic analysis of the cardiac cellulome defines a vascular contribution to cardiac diastolic dysfunction in obese female mice
Source: Basic Res Cardiol. 2023 Mar 29;118(1):11. doi: 10.1007/s00395-023-00983-6 (PMC10060343; doi:10.1007/s00395-023-00983-6)
Supplement: Supplementary file 5 — Supplementary file5 (PDF 465 KB) [file 395_2023_983_MOESM5_ESM.pdf]

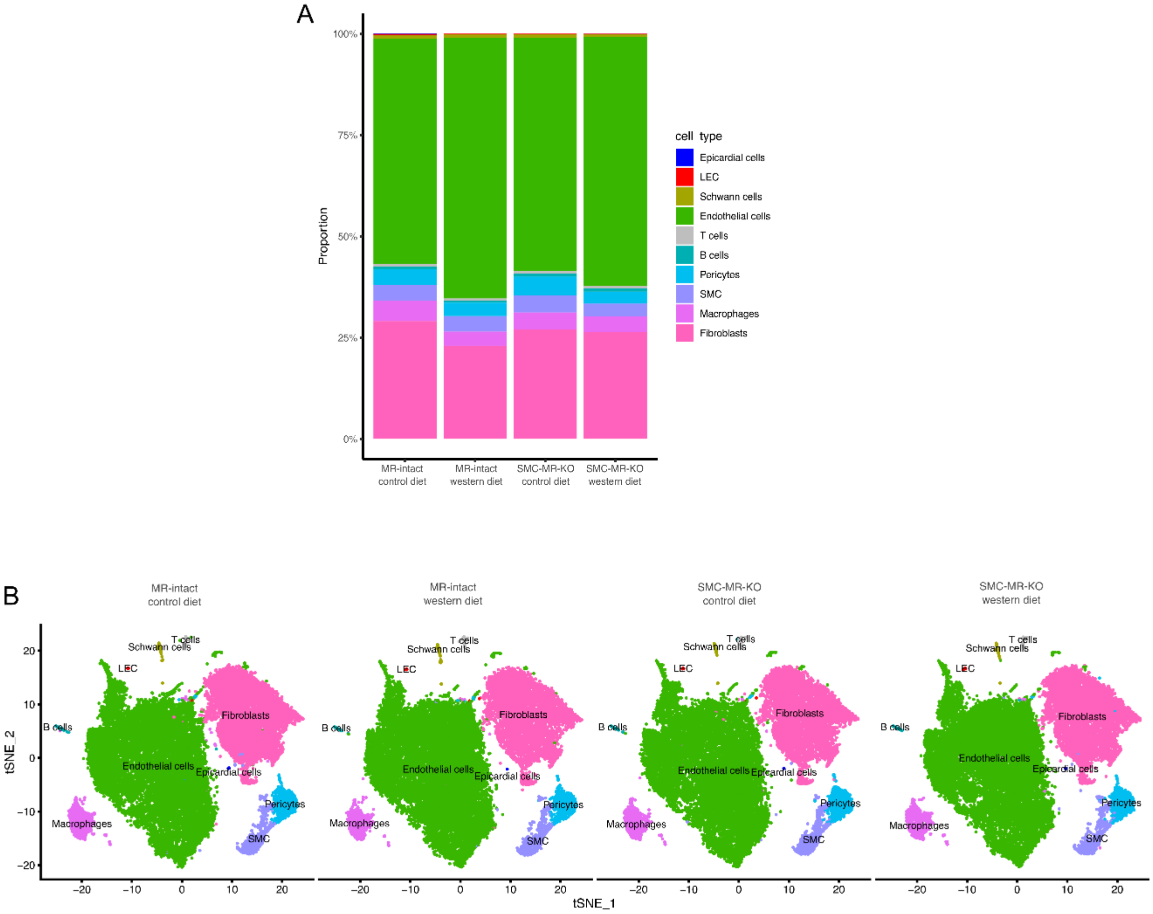

Online Resource 13. *Isolation of cells for single-cell RNA sequencing capture similar proportions of diverse cardiac non-myocyte cell populations across experimental groups.* (**a**) Relative proportions of cell types analyzed from individual samples from mineralocorticoid receptor (MR) Intact and smooth muscle cell MR knockout (SMC-MR-KO) mouse hearts (n=3 per group). Heights of the individual boxes comprising each bar represent the proportion of cell classified as each cell population. (**b**) t-SNE projections of all cardiac non-myocyte cells from each experimental group.

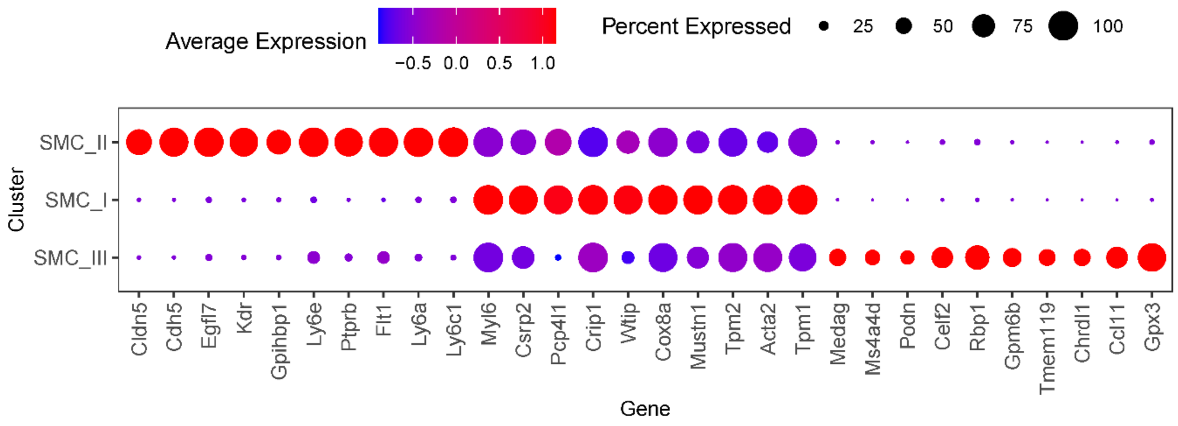

Online Resource 14. *Key gene expression differences between smooth muscle cell (SMC) populations.* Dot plot for top highly and uniquely expressed genes in each smooth muscle cell population identified using an unsupervised analysis. Dot color and size indicate the relative expression and percentage of cells expressing that gene within each cell population, respectively.

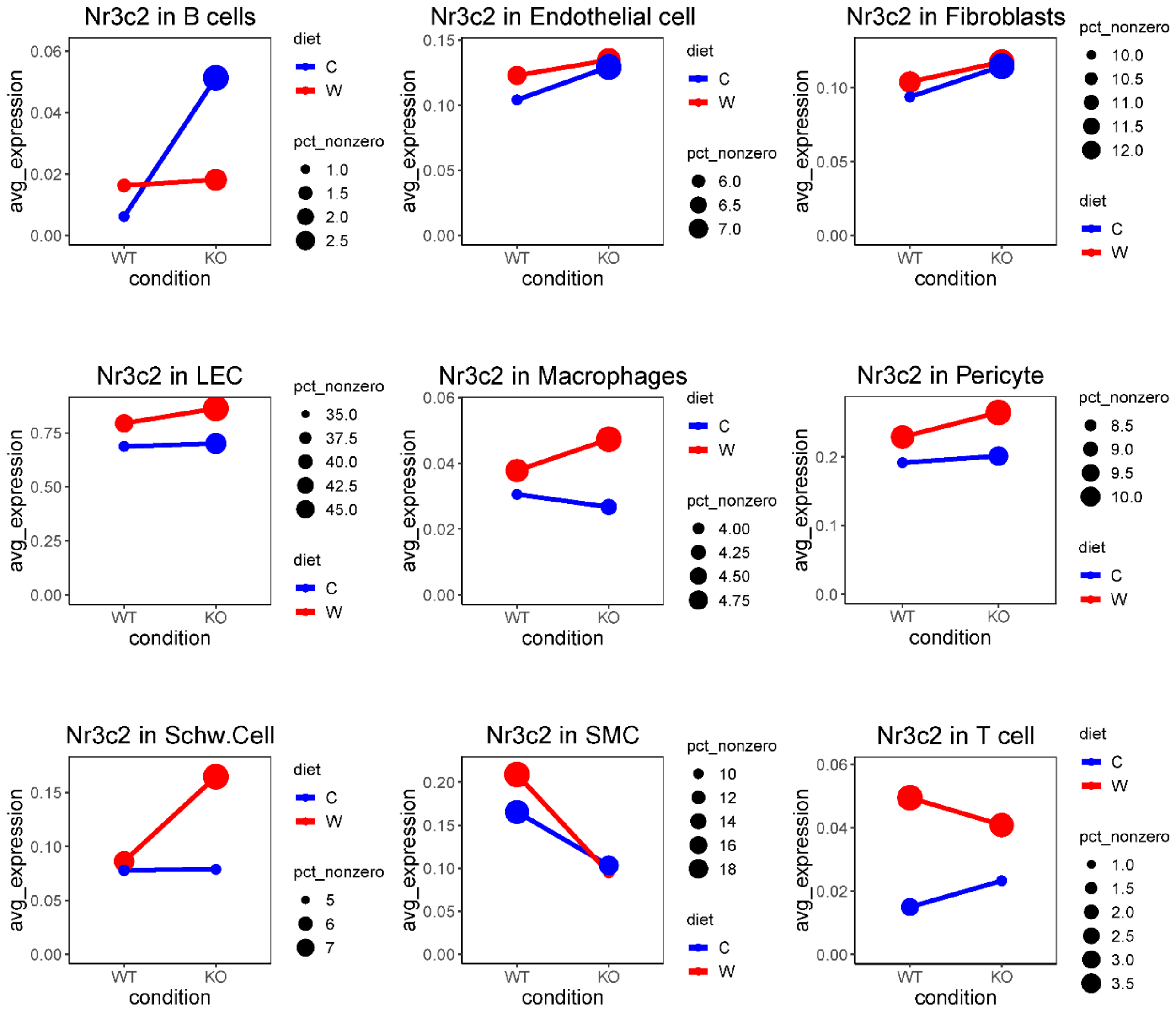

Online Resource 15. *Mineralocorticoid receptor (MR) gene expression in cardiac non-myocyte cell populations with and without western diet.* Gene expression of MR (encoded by *Nr3c2*) in cardiac non-myocytes from control (C) and WD fed (W) MR-intact (WT) and smooth muscle cell MR knockout (KO) mice. Dot color and size indicate the diet group and the percentage of cell expressing *Nr3c2* gene within each group, respectively.
