# Supplementary material for: Multi-omic analysis of the cardiac cellulome defines a vascular contribution to cardiac diastolic dysfunction in obese female mice
Source: Basic Res Cardiol. 2023 Mar 29;118(1):11. doi: 10.1007/s00395-023-00983-6 (PMC10060343; doi:10.1007/s00395-023-00983-6)
Supplement: Supplementary file 7 — Supplementary file7 (PDF 301 KB) [file 395_2023_983_MOESM7_ESM.pdf]

a

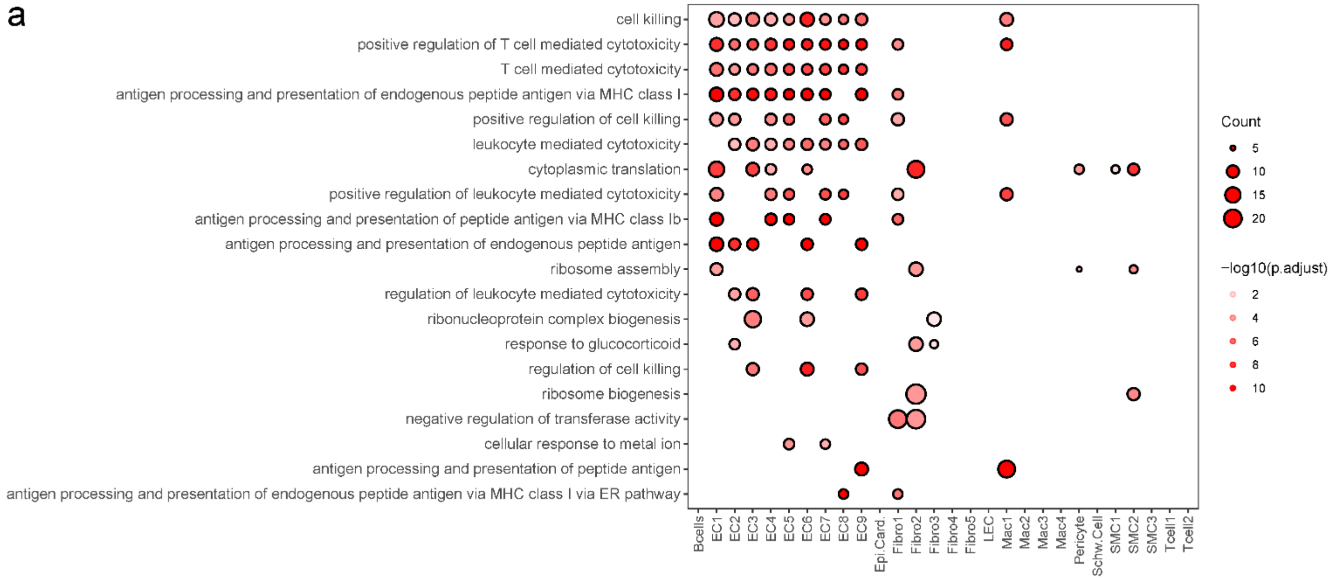

b

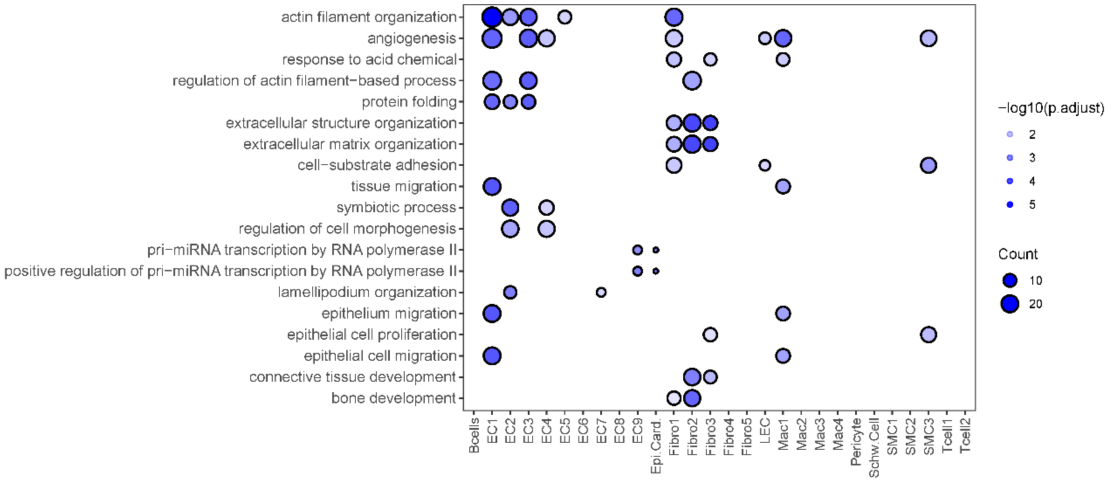

Online Resource 17. *Smooth muscle cell mineralocorticoid receptor knockout (SMC-MR-KO) alters gene programs across cardiac non-myocyte cell populations.* Dot plots summarizing statistically significant gene ontology (GO) terms (corrected $p<0.05$) enriched among genes differentially up- (**a**) or down- (**b**) regulated in each cell population of control diet fed SMC-MR-KO mice versus control diet fed MR-intact mice. Dot color intensity and size indicate significance and number of differentially expressed genes related to each GO term, respectively.
