# Supplementary material for: Multi-omic analysis of the cardiac cellulome defines a vascular contribution to cardiac diastolic dysfunction in obese female mice
Source: Basic Res Cardiol. 2023 Mar 29;118(1):11. doi: 10.1007/s00395-023-00983-6 (PMC10060343; doi:10.1007/s00395-023-00983-6)
Supplement: Supplementary file 10 — Supplementary file10 (PDF 524 KB) [file 395_2023_983_MOESM10_ESM.pdf]

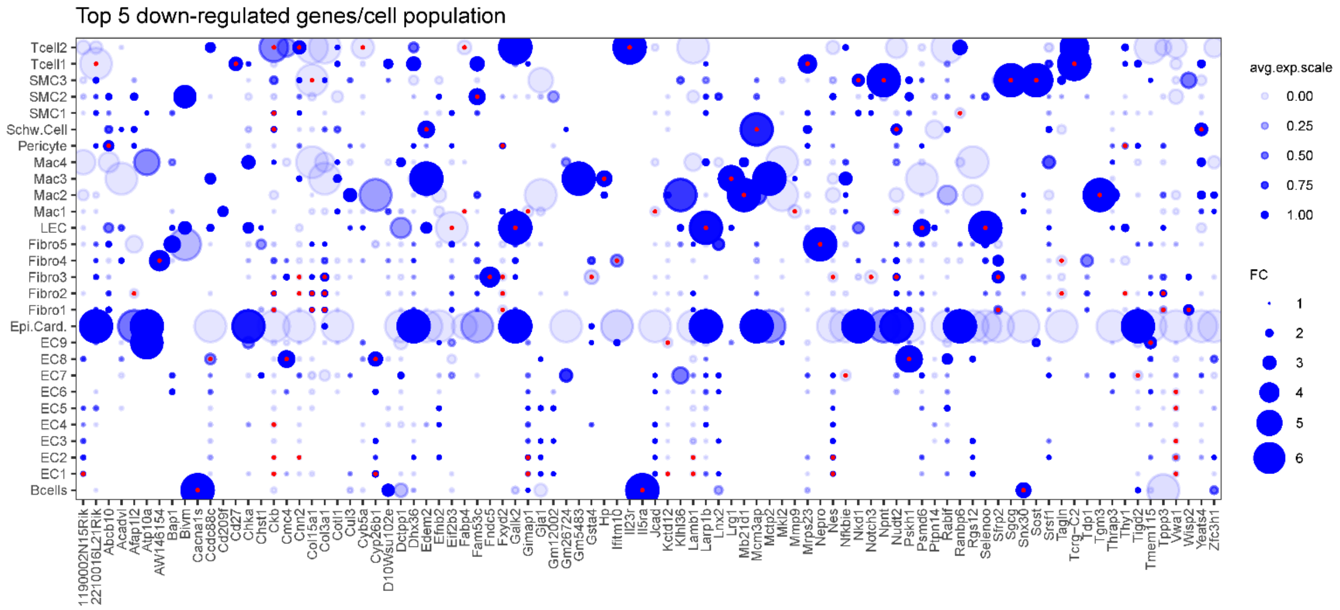

Online Resource 20. *Western diet (WD) feeding down-regulates gene expression in a cell-specific manner in mineralocorticoid receptor (MR) Intact mice.* Dot plot summarizing the relative expression of top significantly down-regulated genes in WD-fed versus control-fed MR-intact mice across cardiac non-myocyte cell populations. Dot color intensity and size are proportional to the relative gene expression in WD cells and the fold change increment in WD cells compared to control cells within each population, respectively. Red points at the centers of some dots highlight statistically significant differences in gene expression in WD cells versus control cells (uncorrected p<0.001).

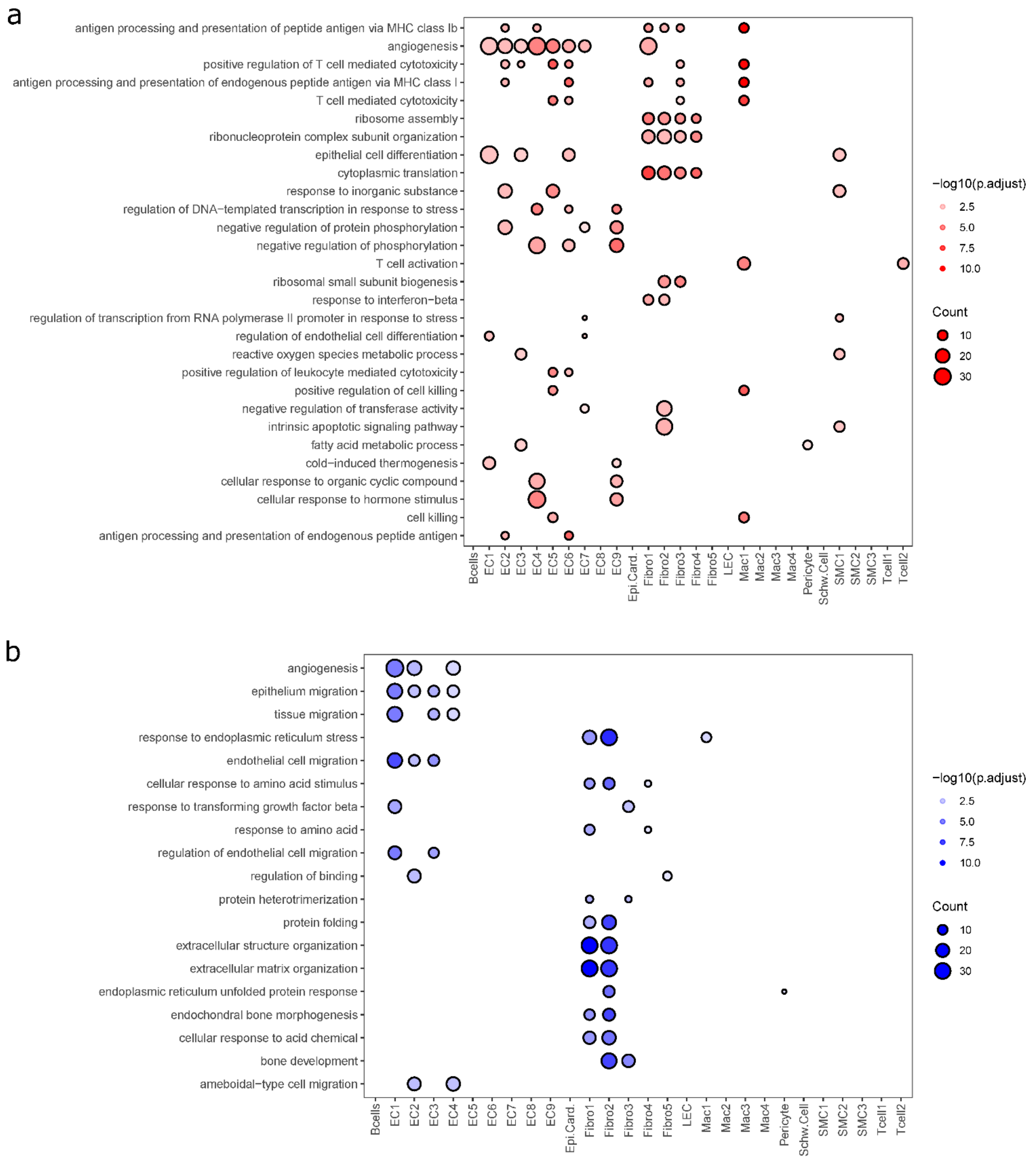

Online Resource 21. *Western diet (WD) feeding impacts gene programs in a cell-specific manner among cardiac non-myocyte populations.* Dot plot summarizing statistically significant gene ontology (GO) terms (corrected $p<0.05$) enriched by WD up- (red dots; **a**) and down-regulated (blue dots; **b**) genes in each cell population from MR-intact mice. Dot color intensity and size indicate significance and number of differentially expressed genes related to each GO term, respectively. GO terms displayed and their order within plots is based on frequency of GO terms in difference cell populations.
