# Supplementary material for: Multi-omic analysis of the cardiac cellulome defines a vascular contribution to cardiac diastolic dysfunction in obese female mice
Source: Basic Res Cardiol. 2023 Mar 29;118(1):11. doi: 10.1007/s00395-023-00983-6 (PMC10060343; doi:10.1007/s00395-023-00983-6)
Supplement: Supplementary file 12 — Supplementary file12 (PDF 929 KB) [file 395_2023_983_MOESM12_ESM.pdf]

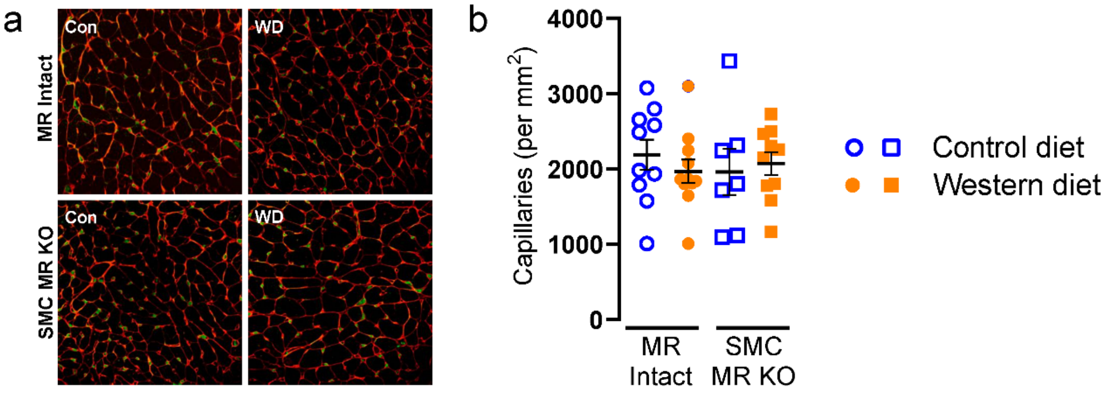

Online Resource 23. *Western diet (WD) or smooth muscle cell mineralocorticoid receptor knockout (SMC-MR-KO) do not change cardiac capillary density.* (**a**) Micrographs of cardiac capillary density, assessed by staining endothelial cells (CD31 staining; red) and nuclei (green), in control- and WD-fed MR-intact and SMC-MR-KO mice. (**b**) Statistical summary of capillary density derived from micrographs. Values are mean±SE with individual data points shown.

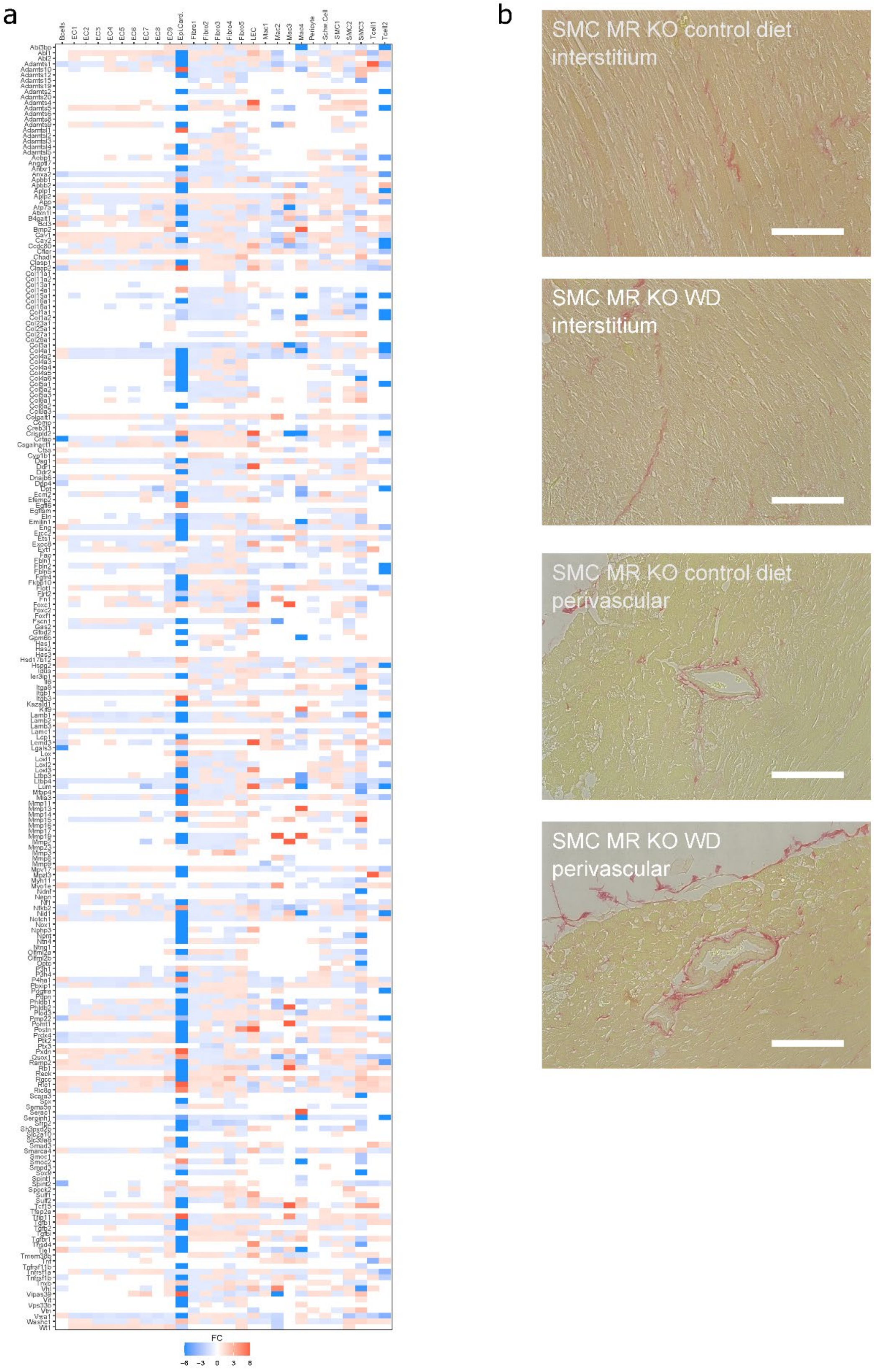

Online Resource 24. *Impact of western diet (WD) feeding on extracellular matrix genes across cardiac non-myocyte cell populations.* (**a**) Heatmap of extracellular matrix genes (from gene ontology term ‘extracellular matrix organization’, #0030198) and their relative expression in MR-intact mice fed either control or WD. Box color and intensity indicate direction: blue indicates down-regulation and red indicates up-regulation of gene after WD. (**b**) Representative interstitial and perivascular fibrosis, assessed by picrosirius red staining, in control- and WD-fed smooth muscle cell mineralocorticoid receptor knockout (SMC-MR-KO) mice.

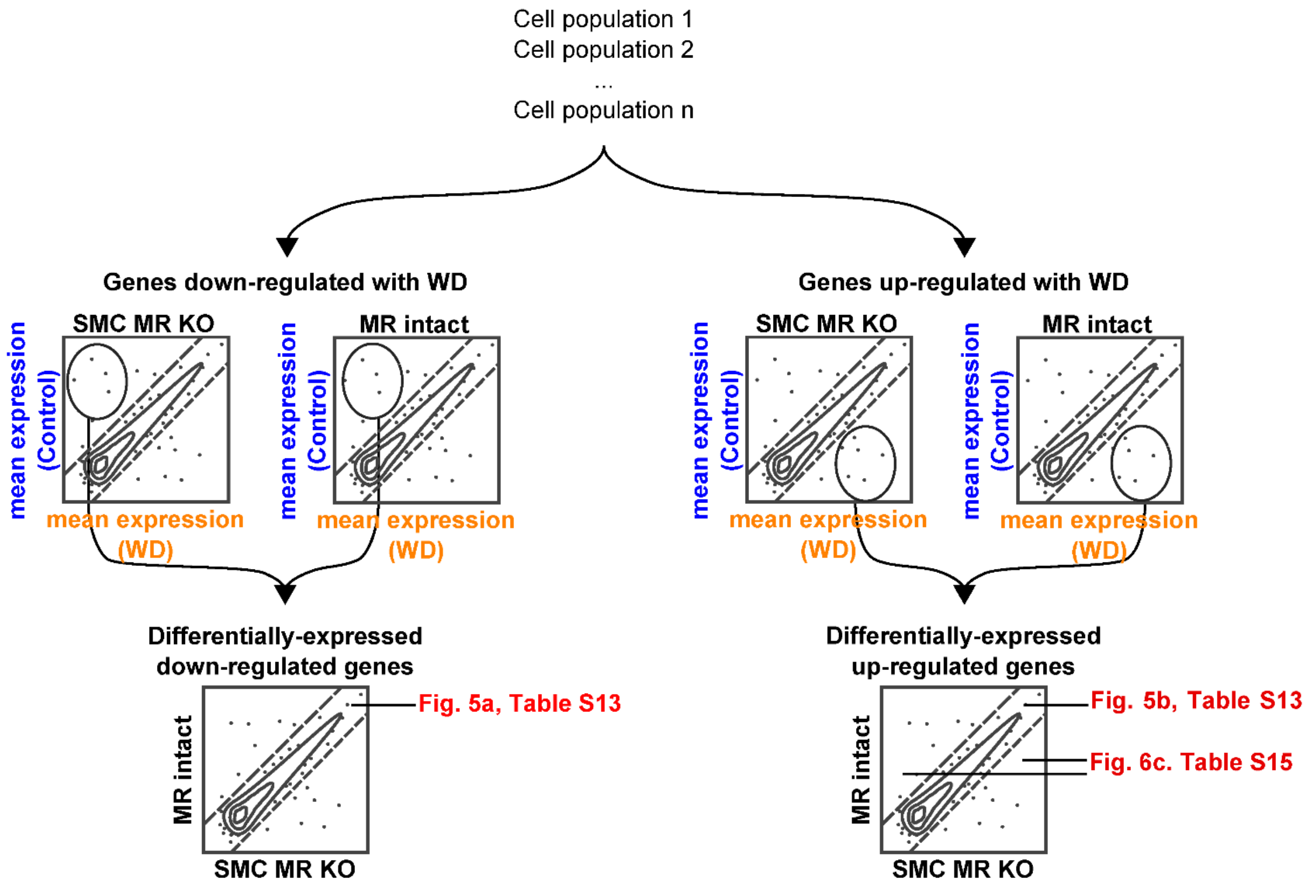

Online Resource 25. *Strategy for determining genes differentially and more robustly impacted by western diet (WD) feeding in mineralocorticoid receptor (MR) Intact versus smooth muscle cell MR knockout (SMC-MR-KO) mice*. For each cell population, MR-Intact and SMC-MR-KO cell populations were considered in isolation for genes upregulated or downregulated after WD. For genotype-specific responses to WD feeding upregulated genes, a list of genes upregulated in either MR-Intact or SMC-MR-KO mice was generated and differences in gene expression examined. For genotype specific responses to WD feeding in downregulated genes, a list of genes downregulated in either genotype was generated and differences in gene expression was again examined.
