# Supplementary material for: Multi-omic analysis of the cardiac cellulome defines a vascular contribution to cardiac diastolic dysfunction in obese female mice
Source: Basic Res Cardiol. 2023 Mar 29;118(1):11. doi: 10.1007/s00395-023-00983-6 (PMC10060343; doi:10.1007/s00395-023-00983-6)
Supplement: Supplementary file 17 — Supplementary file17 (PDF 283 KB) [file 395_2023_983_MOESM17_ESM.pdf]

A

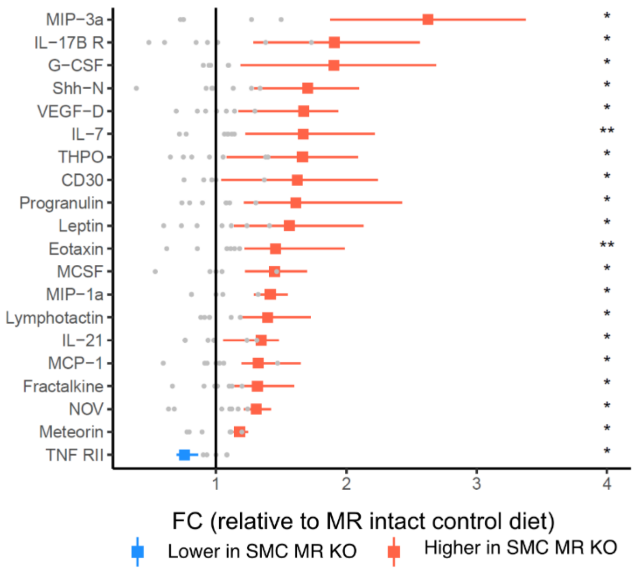

B

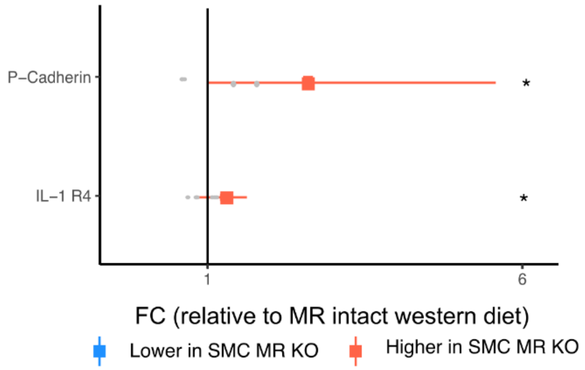

Online Resource 30. *Smooth muscle cell mineralocorticoid receptor knockout (SMC-MR-KO) modulates cardiac cytokines in control diet fed mice.* (**A**) Differentially expressed cardiac cytokines in control diet fed SMC-MR-KO mice versus control diet fed MR-Intact mice. Black line indicates mean cytokine level in MR-intact mouse hearts. (B) Differentially expressed cardiac cytokines in western diet fed SMC-MR-KO mice versus western diet fed MR-Intact mice. Gray dots indicate individual data points for MR-intact group. Red and blue points indicate cytokines at higher or lower levels (respectively) in SMC-MR-KO hearts, relative to mean of MR-intact mouse hearts. Colored squares a indicate mean values with colored lines indicating value range of data points for SMC-MR-KO samples. *p<0.05, **p<0.01.

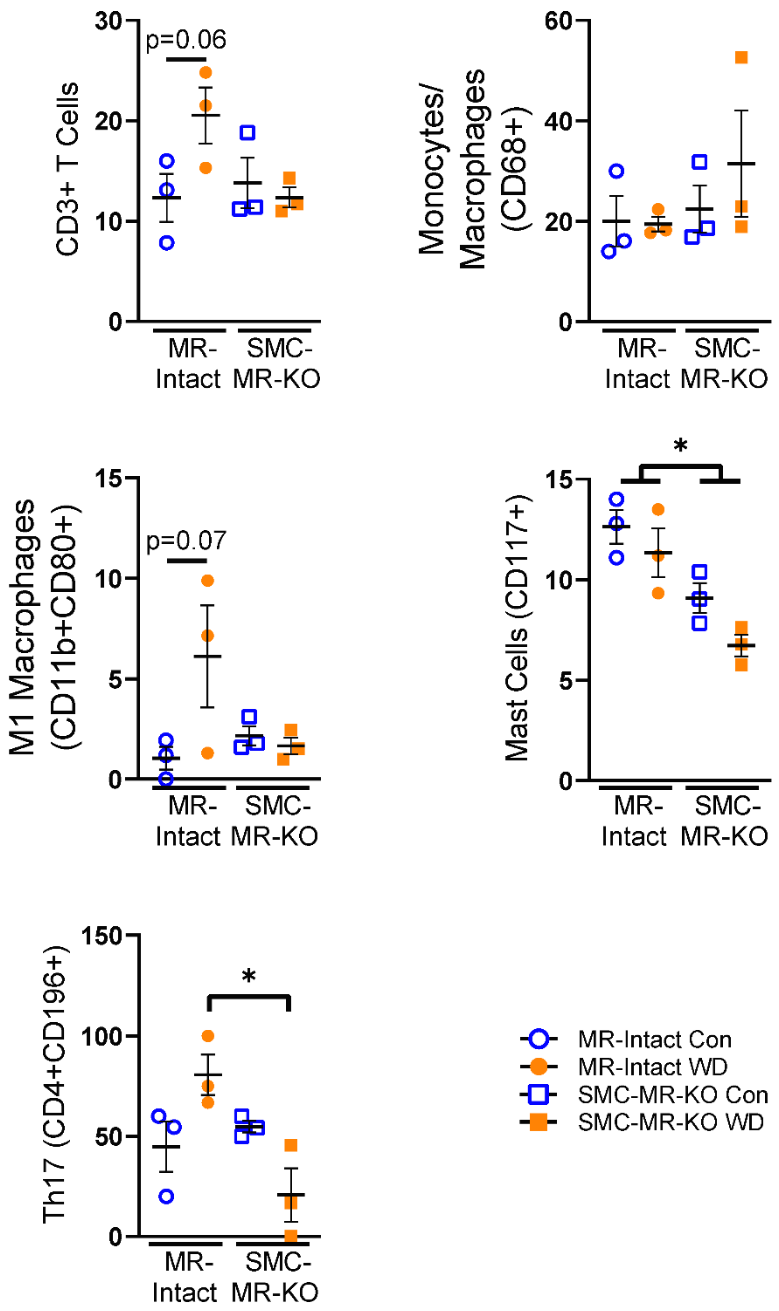

Online Resource 31. *Smooth muscle cell mineralocorticoid receptor knockout (SMC-MR-KO) prevents western diet (WD)-induced cardiac leukocyte recruitment in female mice.* Cardiac leukocytes, specifically CD3+ T cells, CD68+ monocytes/macrophages, CD11b+CD80+ M1 macrophages, CD117+ mast cells, and CD4+CD196+ Th17 cells, assessed by flow cytometry. Values are mean±SE with individual data points shown; *$p<0.05$ for indicated comparison.
